# Synthesis, crystal structure and larvicidal activity of novel diamide derivatives against *Culex pipiens*

**DOI:** 10.1186/1752-153X-6-99

**Published:** 2012-09-11

**Authors:** Rui Wu, Cong Zhu, Xiu-Jiang Du, Li-Xia Xiong, Shu-Jing Yu, Xing-Hai Liu, Zheng-Ming Li, Wei-Guang Zhao

**Affiliations:** 1 State Key Laboratory of Elemento-Organic Chemistry, National Pesticide Engineering Research Center (Tianjin), Nankai University, Tianjin 300071, China; 2 College of Chemical Engineering & Materials Sciences, Zhejiang University of Technology, Hangzhou 310014, China

**Keywords:** Mosquito larvicidal activity, Diamide derivatives, Crystal structure, Synthesis

## Abstract

**Background:**

Culex is an important mosquito as vectors for the transmission of serious diseases, such as filariasis, West Nile virus, dengue, yellow fever, chikungunya and other encephalitides. Nearly one billion people in the developing countries are at risk. In order to discover new bioactive molecules and pesticides acting on mosquito, we designed active amide structure and synthesized a series of novel diamide derivatives.

**Results:**

A series of novel diamide derivatives were designed and synthesized. Their structures were characterized by ^1^ H NMR, FTIR and HRMS. The single crystal structure of compound 6n was determined to further elucidate the structure. Biological activities of these compounds were tested. Most of them exhibited higher mosquito larvicidal activity. Especially compound 6r displayed relatively good activity to reach 70% at 2 μg/mL.

**Conclusion:**

A practical synthetic route to amide derivatives by the reaction of amide with another acid is presented. This study suggests that the diamide derivatives exhibited good effective against mosquito.

## Background

Culex is an important mosquito as vectors for the transmission of serious diseases [1,2], such as filariasis, West Nile virus, dengue, yellow fever, chikungunya and other encephalitides. Lymphatic filariasis, which may be caused by different species of filarial worm, e.g., *Wuchereria bancrofti*, has a scattered distribution in the tropics and subtropics [3]. Nearly one billion people in the developing countries are at risk. *Culex pipiens L.* is the most commonly occurring mosquito pest in urban and suburban areas [4], which is mainly the intermediate host and vector of *Bancroftian filariasis*.

According to World Health Organisation (WHO), the one of strategies is to destroy their vectors or intermediate hosts. The best method is control of mosquito larvae using insecticides [5-7], such as organo-phosphates, natural products and heterocycles types. It is an urgent need to develop new insecticides which are more environmentally safe and also biodegradable and target specific against mosquitoes.

Amide derivatives have become one of the focuses in the development of pesticides because of their high biological activities, such as fungicidal activities [8-11], herbicidal activities [12-15], insecticidal activities [16,17], anticancer activity [18,19], antibacterial activity [20] and so on. In line with our continueous efforts to synthesize bioactive lead compounds, the title compounds were designed by introducing amide pharmacophore into the valine scaffold. Thus, guaiacol was used as start materials, 22 novel diamides were synthesized. All the compounds were unequivocally characterized by NMR, IR, HRMS. The single crystal structure of compound 6n was determined to further elucidate the structure. The biological activities of title compounds against *Culex pipiens* were determined, the results showed that most of the synthesized compounds exhibited antibacterial activity against *Culex pipiens*, compounds 6n showed good activity against *Culex pipiens* at 2 μg/mL.

## Results and discussion

### Synthesis and spectra

**Scheme 1:**
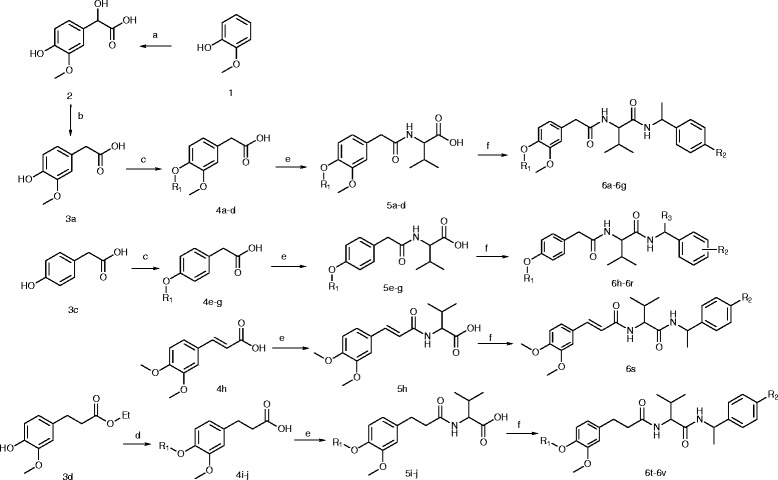
The Synthesis Route of title compounds.

The synthetic route to the title compounds is outlined in Scheme 1 [see Additional file 1]. Compound 3 was synthesized at the condition of neutral. When the 2-oxoacetic acid was drop wised to the start material 2-methoxyphenol, the pH of the mixture increase, while it must be reacted under pH = 7, as higher pH decreased the yield of product. So a solution of NaOH should be drop wised at the same time. Several procedures are available for the synthesis of amide derivatives. Due to the dissolve of valine in the organic solvent, such as THF, so it can not react with the 2-phenylacetyl chloride. In this paper, the 2-phenylacetyl chloride was reacted with the solution of sodium 2-amino-3-methylbutanoate in water.

The structures of all new compounds were confirmed by their spectra (^1^ H NMR and FTIR) data. Additional file shows the structures, yields and HRMS data for title compounds in more detail [see Additional file 2]. The proton magnetic resonance spectra of the amides have been recorded in CDCl_3_. The COOH of amide intermediates 4 is not determined. All the title compounds of HRMS are M + H, or M + Na peak.

### Crystal structure

**Figure 1:**
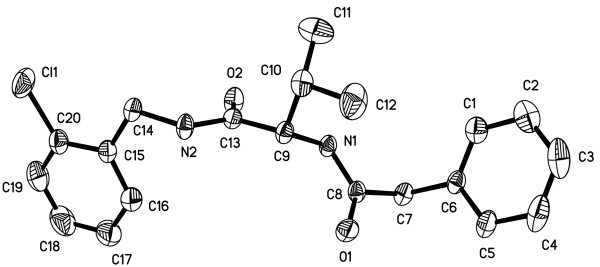
The crystal structure of compound 6n.

The structure of compound 6n was further confirmed by single crystal X-ray diffraction analysis (Figure 1). Generally, the average bond lengths and bond angles of phenyl ring and amide bond are normal ranges. The C8-N1 and C13-N2 bond [1.337(2) Å and 1.334(2) Å] is shorter than a normal C-N single bond (1.47 Å), which shows that C8-N1 and C13-N2 is conjugated with the O1-C8 and O2-C13 double bond respectively. The sum of the C–N–C angles around the N atom is 122º which means in the solid state there is no p-π conjugation between the benzyl group and the N atom. In the molecular structure of compound 6n, acyl group is planar with amide group and the torsional angles of O1-C8-N1-H1 and O2-C13-N2-H2 is 178.68^o^ and 177.06^o^. The C1-C6 and C15-C20 phenyl rings are fairly planar with plane equation 5.176x + 3.771y – 6.693z = 2.7161 and 9.008x + 4.192y – 0.151z = 3.0785, the mean deviation from the plane is 0.0026 and 0.0070 Å respectively. In the molecular structure of title compound, the θ angle of the two benzene rings is 39.7º, and the distance is 10.68 Å between the two rings. Moreover, intermolecular N1-H1-O2 hydrogen bonds are also observed in the crystal structure. The title compound has an extensive one dimension chain polymer of hydrogen bonding involving the atoms, N and O. In the *bc* plane, they are linked together by N1-H1-O2 hydrogen bonds. This hydrogen-bonding sequence is repeated to form a ring. The ring is shaped like a decagon and has two O1 atoms at the vertices, leading to a hydrogen-bond network defining cyclic motifs denoted $R_{2}^{2}$(10). The vertices are shared with neighboring decagon to form an infinite two-dimensional network of hydrogen bonds in the *bc* plane.

### Larvicidal activity against mosquito

The toxicity of test compounds to *Culex pipiens pallens* larvae is listed in Table 1. The results indicated that most of the title compounds exhibited excellent activities against mosquito at 5 μg/mL. For example, the larvicidal activities of compounds 6b, 6 m, 6p, 6r and 6 t against mosquito at 5 μg/mL are 70%, 80%, 70%, 100% and 70%, respectively. Especially, compound 6r still maintain high insecticidal activity (70%), even at 2 μg/mL. All the title compounds exhibited moderate larvacidal activities against mosquito, except compound 6j.

**Table 1:** Larvicidal activity against Mosquito of title compounds at 5 μg/mL

**No.**	**Death rate (%)**	**No.**	**Death rate (%)**
**6a**	50	**6 l**	50
**6b**	70	**6 m**	80
**6c**	50	**6n**	50
**6d**	53	**6o**	50
**6e**	53	**6p**	70
**6f**	50	**5q**	60
**6 g**	43	**6r**	100
**6 h**	54	**6 s**	50
**6i**	60	**6 t**	70
**6j**	30	**6u**	43
**6 k**	50	**6v**	67

### Experimental

#### Chemistry

Melting points were determined by an X-4 apparatus and uncorrected. ^1^ H NMR spectra were measured on a Bruker AV-400 instrument using TMS as an internal standard and DMSO-*d*_6_ as the solvent. HRMS data was obtained on a FTICR-MS instrument (Ionspec 7.0 T). Crystallographic data of the compound were collected on a rigaku saturn diffractometer. Microwave activation was carried out with CEM Discover™ focused microwave (2450 MHz, 300 W). All the reagents are of analytical grade or freshly prepared before use. The course of the reactions was monitored by TLC; analytical TLC was performed on silica gel GF 254. Intermediates 2, 3, 4 and 5 were prepared according to the reported methods [21-29] and used without further purifications, the process for preparing of them can be found in Additional file 2.

#### Crystal structure

The crystal of compound 6n with dimensions of 0.20 mm × 0.18 mm × 0.14 mm was mounted on a Rigaku Saturn CCD area-detector diffractometer with a graphite-monochromated MoKα radiation (λ = 0.71073 Å) by using a phi and scan modes at 293(2) K in the range of 2.55° ≤ θ ≤ 27.85°. The crystal belongs to Triclinic system with space group P-1 and crystal parameters of a = 9.4583(19) Å, b = 10.494(2) Å, c = 11.545(2) Å, α = 70.49(3)°, β = 73.27(3)°, γ = 64.00(3)°, V = 957.2(3)A^3^,Dc = 1.245 g/cm^3^. The absorption coefficient μ = 0.0815 mm^-1^ and Z = 2. The structure was solved by direct methods with SHELXS-97 and refined by the full-matrix least squares method on F^2^ data using SHELXL-97 [30]. The empirical absorption corrections were applied to all intensity data. H atom of N-H was initially located in a difference Fourier map and were refined with the restraint Uiso(H) = 1.2Ueq(N). Other H atoms were positioned geometrically and refined using a riding model, with d(C---H) = 0.93-0.97 Å and Uiso(H) = 1.2Ueq(C) or 1.5Ueq(Cmethyl). The final full-matrix least squares refinement gave R = 0.0476 and *wR*^*2*^ = 0.0971.

#### Larvicidal activity against mosquito

A stock solution of each compound was prepared at 1000 μg/mL using acetone as a solvent. Each compound in acetone was suspended in distilled water with Tween-80 (0.001%). Distilled water mixed with Tween-80 was used as control. Batches of 10 fourth-stage larvae of C. pipiens pallens were separately put into Beakers (100 mL) containing each test solution (40 mL) using a pipet. Each test compound was evaluated at the level of 2 μg/mL in distilled water. Observation on larval mortality was recorded after 24 h. The larvae were considered dead if appendages did not move when prodded with a needle. The experimental results are summarized in Table 1.

$$\text{Rectified mortality}\;\left(\%\right)\;=\;\left(A1-A2\right)/\left(100-A2\right)\times 100$$

where the Al (%) is the mortality in treatment group, and the A2 (%) is the mortality in control group.

## Conclusion

In summary, a novel series of diamide derivatives were designed and synthesized. The synthesized compounds were characterized by spectral data (^1^ H NMR) and HRMS (ESI). All of the compounds were subjected to larvicidal activity against mosquito*.* The results indicated that the synthesized compounds possessed good larvicidal activity against mosquito. Further studies are currently underway to optimize to enhance the larvicidal activity of the diamide derivatives.

## Competing interests

The authors declare that they have no competing interests.

## Authors’ contributions

The current study is an outcome of constructive discussion with XHL, ZML and WGZ who offered necessary guidance to RW and RW to carry out their synthesis and characterization experiments. XHL were also involved in the drafting of the manuscript. LXX and SJY performed the biological activity tests; RW, CZ, XJD carried out the ^1^ H NMR and HRMS, XHL elucidate the single crystal. WGZ were involved in revising the manuscript. All authors read and approved the final manuscript.

## Supplementary Material

Additional file 1 Supporting information. Click here for file

Additional file 2 Contains the CIF for compound 1. Click here for file
