# Supplementary material for: Synthesis, crystal structure and larvicidal activity of novel diamide derivatives against Culex pipiens
Source: Chem Cent J. 2012 Sep 11;6:99. doi: 10.1186/1752-153X-6-99 (PMC3537643; doi:10.1186/1752-153X-6-99)

Additional file 2

**Synthesis, Crystal Structure and Larvicidal Activity of Novel Diamide Derivatives against *Culex pipiens***

Rui Wu^1^, Cong Zhu^1^, Xiu-Jiang Du^1^, Li-xia Xiong^1^, Shu-Jing Yu^1^, Xing-Hai Liu^2*^, Zheng-Ming Li^1^, Wei-Guang Zhao^1*^

Address: *1. State Key Laboratory of Elemento-Organic Chemistry, National Pesticide Engineering Research Center(Tianjin), Nankai University, Tianjin 300071, China*

*2. College of Chemical Engineering & Materials Sciences, Zhejiang University of Technology, Hangzhou, 310014, China*

Author to whom correspondence should be addressed;

Tel.: *Corresponding author Xing-Hai Liu. Tel: +86 571 88320147; Fax: +86 571 88320147; Wei-Guang Zhao. Tel: +86 22 23500074 Fax: +86 22 23500074;

E-Mail: WR: [wurui08@mail.nankai.edu.cn](mailto:wurui08@mail.nankai.edu.cn)

CZ: [zhucong@mail.nankai.edu.cn](mailto:zhucong@mail.nankai.edu.cn)

XJD: [dxj881216@126.com](mailto:dxj881216@126.com)

LXX: [xionglixia@nankai.edu.cn](mailto:xionglixia@nankai.edu.cn)

SJY: [yushujing@nankai.edu.cn](mailto:yushujing@nankai.edu.cn)

XHL: [xhliu@zjut.edu.cn](mailto:xhliu@zjut.edu.cn)

ZML: [zml@nankai.edu.cn](mailto:zml@nankai.edu.cn)

WGZ: [zwg@nankai.edu.cn](mailto:zwg@nankai.edu.cn)

**Supporting Information**

### Table contents

Experimental procedureS1

The physical and spectral data S1

Copies of ^1^H NMR and HRMS, FT-IR S13

Synthesis of Intermediates

The key intermediate 2-hydroxy-2-(4-hydroxy-3-methoxyphenyl)acetic acid **2** was synthesized according the references. M.p. 130-131 ^o^C(ref. 131~132 ^o^C).

To the intermediate **2** (24 mmol), was added a solution of tin(II) chloride (8.10 g, 36 mmol) in concentrated hydrochloric acid (20 mL). The reaction mixture was stirred at 83-85 ^o^C for 24 h. After cooling, a light yellow precipitate was collected to give compound 2-(4-hydroxy-3-methoxyphenyl)acetic acid **3a**. Yield 84%, m.p. 130~134 ^o^C. (ref.：138~140 ^o^C).

General process for **4a**

To a stirred solution of **3a** (1.68 g, 10 mmol) in methanol(10 mL), TBAB (0.32 g, 1.0 mmol) was added a solution of NaOH (1.00 g, 25 mmol) in H_2_O (10 mL) at 0 ^o^C. The mixture was stirred for 0.5 h. Then bromoethane (12 mmol) was dropwised. The resulting mixture was stirred at room temperature for overnight. Then diluted with ethyl acetate (20 mL), and washed with 1 mol•L^-1^ aq. HCl solution (10 mL), and extracted several times with ethyl acetate. The combined organic phases were washed with brine, dried over MgSO_4_ and evaporated. The remainder was purified by chromatography on silica gel using petroleum ether (60-90 ^o^C) and ethyl acetate as the eluent to afford the compounds **4a.** Compounds **4b-4i** were synthesized using the same process as compound **4a**.

**2-(4-ethoxy-3-methoxyphenyl)acetic acid 4a**: white solid, m.p. 100~103 ^o^C (literature：183~185 ^o^C(Barger et al., 1933)), yield 66%.

**2-(3-methoxy-4-propoxyphenyl)acetic acid 4b**: white solid, m.p. 85~87 ^o^C (literature：87~88 ^o^C(Shepard et al., 1952)).

**2-(4-(allyloxy)-3-methoxyphenyl)acetic acid 4c**: white solid, m.p. 117~119^o^C, yield 68%; ^1^H NMR(400 MHz, CDCl_3_) : δ 6.88-6.81 (m, 3H, Ar), 6.14-6.07 (m, 1H, OCH_2_CH=CH_2_), 5.43 (d, *J =* 17.2Hz, 1H, OCH_2_CH=CH^a^H^b^), 5.31 (d, *J =* 10.2Hz, 1H, OCH_2_CH=CH^a^H^b^), 4.63 (s, 2H, OCH_2_CH=CH_2_), 3.90 (s, 3H, OCH_3_), 3.63 (s, 2H, CH_2_COOH) 4.70 (s, 2H, OCH_2_CH), 3.62 (s, 2H, CH_2_COOH), 2.55 (s, 1H, OCH_2_CH).

**2-(3-methoxy-4-(prop-2-yn-1-yloxy)phenyl)acetic acid 4d**: white solid, m.p. 103~106^o^C, yield 65%; ^1^H NMR(400 MHz, CDCl_3_) : δ 6.89-6.82 (m, 3H, Ar), 4.70 (s, 2H, OCH_2_CCH), 3.89 (s, 3H, OCH_3_), 3.61 (s, 2H, CH_2_COOH), 2.55 (s, 1H, OCH_2_CCH), 1.91-1.86 (m, 2H, OCH_2_CH_2_CH_3_).

**2-(4-(prop-2-yn-1-yloxy)phenyl)acetic acid 4i**: white solid, m.p. 60-64^o^C, yield 94%; ^1^H NMR (400 MHz, CDCl_3_): δ 7.24 (d, *J =* 7.9Hz, 2H, Ar-H), 6.97 (d, *J =* 7.8Hz, 2H, Ar-H), 4.70 (s, 2H, OCH_2_CCH), 3.62 (s, 2H, CH_2_COOH), 2.55 (s, 1H, OCH_2_CCH).

**3-(3-methoxy-4-(prop-2-yn-1-yloxy)phenyl)propanoic acid 4j:**, white solid, yield 98%; ^1^H NMR (400 MHz, CDCl_3_): δ 6.98 (d, *J =* 8.0Hz, 1H, Ar-H), 6.78-6.73 (m, 2H, Ar-H), 4.75 (s, 2H, OCH_2_CCH), 3.88 (s, 3H, OCH_3_), 2.93 (t, *J =* 7.7Hz, 2H, CH_2_CH_2_COOH), 2.69 (t, *J =* 7.7Hz, 2H, CH_2_CH_2_COOH), 2.52 (s, 1H, OCH_2_CCH).

Compound **4e** (2.04 g, 15 mmol) in dichloromethane (20 mL) was added oxalyl chloride (2.86 g, 22.5 mmol), followed by dimethylformamide (2 drops). The solution was stirred at room temperature. After 24 h the mixture was concentrated *in vacuo* to obtain the crude acid chloride. The crude acid chloride in dichloromethane (10 mL) was added slowly to a stirred solution of sodium 2-amino-3-methylbutanoate (2.11 g, 18 mmol) in water (20 mL) in an ice bath. The solution was warmed to room temperature and stirred for 10 h. The mixture was acid to pH= 1 by dilute HCl, white precipitate was collected to give compound **5e**. The other compounds **5** were synthesized using the same process as compound **5e**.

**2-(2-(4-ethoxy-3-methoxyphenyl)acetamido)-3-methylbutanoic acid (5a)**: white solid, m.p. 97~98^o^C, yield 84%; ^1^H NMR(400 MHz，DMSO-d_6_): δ 8.19 (d, *J =* 8.5Hz, 1H, NH), 6.92 (s, 1H, Ar-H), 6.84 (d, *J =* 8.1Hz, 1H, Ar-H), 6.75 (d, *J =* 8.2Hz, 1H, Ar-H), 4.15-4.12 (m, 1H, NHCHCOOH), 3.96 (q, *J =* 6.9Hz, 2H, OCH_2_CH_3_), 3.72 (s, 3H, OCH_3_), 2.09-2.01 (m, 1H, (CH_3_)_2_CH), 1.29 (t, *J =* 6.9Hz, 3H, OCH_2_CH_3_), 0.87 (dd, *J*_1_ = 6.8Hz, *J*_2_ = 11.6Hz, 6H, (CH_3_)_2_CH).

**2-(2-(3-methoxy-4-propoxyphenyl)acetamido)-3-methylbutanoic acid (5b)**: white solid, m.p. 132~136^o^C, yield 90%; ^1^H NMR(400 MHz，CDCl_3_): δ 6.89 (d, *J =* 7.5Hz, 1H, Ar-H), 6.82 (s, 2H, Ar-H), 6.01 (t, *J =* 8.5Hz, 1H, NH), 4.56-4.54 (m, 1H, NHCHCOOH), 4.00 (t, *J =* 6.5Hz, 2H, OCH_2_CH_2_CH_3_), 3.88 (s, 3H, OCH_3_), 3.61 (s, 2H, CH_2_CO), 2.21-2.19 (m, 1H, (CH_3_)_2_CH), 1.94-1.85 (m, 2H, OCH_2_CH_2_CH_3_), 1.07 (t, *J =* 7.3Hz, 3H, OCH_2_CH_2_CH_3_), 0.92 (dd, *J =* 6.5Hz, 3H, (CH_3_)_2_CH), 0.81 (dd, *J =* 6.5Hz, 3H, (CH_3_)_2_CH).

**2-(2-(4-(allyloxy)-3-methoxyphenyl)acetamido)-3-methylbutanoic acid (5c)**: white solid, m.p. 117~119^o^C, yield 50%; ^1^H NMR(400 MHz，CDCl_3_): δ 6.90-6.75 (m, 3H, Ar-H), 6. 14-6.18 (m, 1H, OCH_2_CH=CH_2_), 6.00 (d, *J =* 8.3Hz, 1H, NH), 5.43 (d, *J =* 17.3Hz, 1H, OCH_2_CH=CH^a^H^b^), 5.32 (d, *J =* 10.3Hz, 1H, OCH_2_CH=CH^a^H^b^), 4.64 (s, 2H, OCH_2_CH=CH_2_), 4.55 (t, *J =* 4.3Hz, 1H, NHCHCOOH), 3.89 (s, 3H, OCH_3_), 3.61 (s, 2H, CH_2_CO), 2.26-2.19 (m, 1H, (CH_3_)_2_CH), 0.92 (dd, *J =* 6.1Hz, 3H, (CH_3_)_2_CH), 0.81 (dd, *J =* 6.1Hz, 3H, (CH_3_)_2_CH).

**2-(2-(3-methoxy-4-(prop-2-yn-1-yloxy)phenyl)acetamido)-3-methylbutanoic acid (5d)**: Brown solid, m.p. 103~105^o^C, yield 81%; ^1^H NMR(400 MHz，CDCl_3_) : δ 6.94 (d, *J =* 8.0Hz, 1H, Ar-H), 6.81 (s, 1H, Ar-H), 6.76 (d, *J =* 8.1Hz, 1H, Ar-H), 6.37 (d, *J =* 8.5Hz, 1H, NH), 4.69 (s, 2H, OCH_2_CCH), 4.50 (dd, *J*_1_ = 4.3Hz, *J*_2_ = 7.9Hz, 1H, NHCHCOOH), 3.79 (s, 3H, OCH_3_), 3.55 (s, 2H, CH_2_CO), 2.49 (s, 1H, OCH_2_CCH), 0.84 (d, *J =* 6.6Hz, 3H, (CH_3_)_2_CH), 0.81 (d, *J =* 6.7Hz, 3H, (CH_3_)_2_CH).

**3-methyl-2-(2-phenylacetamido)butanoic acid (5e)**: white solid, m.p. 136~138 ^o^C (literature: 137~138^o^C(Buu-Hoi et al., 1969)), yield 84%.

**2-(2-(4-chlorophenyl)acetamido)-3-methylbutanoic acid (5f)**: white solid, m.p. 169~170 ^o^C (literature:144~145^o^C(Otto et al., 1949)), yield 90%.

**3-methyl-2-(2-(4-(prop-2-yn-1-yloxy)phenyl)acetamido)butanoic acid (5g)**: Brown solid, m.p. 103~105^o^C, yield 81%; ^1^H NMR(400 MHz，CDCl_3_): δ 8.10 (d, *J =* 8.6Hz, NH), 7.24 (d, *J =*  7.9Hz, 2H, Ar-H), 6.97 (d, *J =* 7.8Hz, 2H, Ar-H), 4.14-410 (m, 1H, NHCHCOOH), 3.38 (d, *J =* 17.2Hz, 2H, CH_2_CONH), 2.55 (s, 1H, OCH2CCH), 2.08-2.01 (m, 1H, (CH_3_)_2_CH), 0.87 (dd, *J*_1_ = 6.9Hz, *J*_2_ = 9.7Hz, 6H, (CH_3_)_2_CH).

**(E)-2-(3-(3,4-dimethoxyphenyl)acrylamido)-3-methylbutanoic acid (5h)**: white solid, m.p.165~167^o^C, yield 50%; ^1^H NMR(400 MHz，DMSO-d_6_) : δ 8.00 (d, *J =* 8.6Hz, 1H, NH), 7.55 (m, 1H, CH=CHCO), 6.84-6.70 (m, 4H, CH=CHCO, Ar-H), 4.16-4.12 (m, 1H, NHCHCOOH), 3.72 (s, 3H, OCH_3_), 3.71 (s, 3H, OCH_3_), 2.05-1.96 (m, 1H, (CH_3_)_2_CH), 0.83 (t, *J =* 6.3Hz, 6H, (CH_3_)_2_CH).

**2-(3-(3,4-dimethoxyphenyl)propanamido)-3-methylbutanoic acid (5i)**: white solid, m.p. 132~136^o^C, yield 53%; ^1^H NMR(400 MHz，DMSO-d_6_) : δ 7.99 (d, *J =* 8.6Hz, 1H, NH), 6.82 (d, *J =* 8.5Hz, 2H, Ar-H), 6.71 (d, *J =* 8.1Hz, 1H, Ar-H), 4.16-4.12 (m, 1H, NHCHCOOH), 3.72 (s, 3H, OCH_3_), 3.70 (s, 3H, OCH_3_), 2.74 (t, *J =* 7.7Hz, 2H, CH_2_CH_2_CO), 2.48-2.42 (m, 2H, CH_2_CH_2_CO), 2.05-1.96 (m, 1H, (CH_3_)_2_CH), 0.83 (t, *J =* 6.3Hz, 6H, (CH_3_)_2_CH).

**2-(3-(3-methoxy-4-(prop-2-yn-1-yloxy)phenyl)propanamido)-3-methylbutanoic acid (5j)**: white solid, m.p. 148~149^o^C, yield 56%; ^1^H NMR(400 MHz，CDCl_3_) : δ 6.98 (d, *J =* 7.8Hz, 1H, Ar-H), 6.77 (d, *J =* 8.8Hz, 2H, Ar-H), 5.87 (d, *J =* 8.4Hz, 1H, NH), 4.75 (s, 2H, OCH_2_CCH), 4.56 (dd, *J*_1_ = 5.2Hz, *J*_2_ = 8.1Hz, 1H, NHCHCOOH), 3.88 (s, 3H, OCH_3_), 2.96 (t, *J =* 7.2Hz, 2H, CH_2_CH_2_CO), 2.63-2.55 (m, 2H, CH_2_CH_2_CO), 2.51 (s, 1H, OCH_2_CCH), 2.22-2.17 (m, 1H, (CH_3_)_2_CH), 0.92 (d, *J =* 6.8Hz, 3H, (CH_3_)_2_CH), 0.89 (d, *J =* 6.8Hz, 3H, (CH_3_)_2_CH).

***2.2 General method for preparing title compounds***:

To a mixture of 3-methyl-2-(2-substituted phenylacetamido)butanoic acid **5** (2.5 mmol) and Et_3_N (0.28 g，2.75 mmol) in CH_2_Cl_2_ (10 mL) was added isobutyl chloroformate (0.34 g，2.5 mmol). The mixture was stirred for 1 h, then a solution of substituted benzylamine (0.38 g，2.75 mmol) in CH_2_Cl_2_ (10 mL) was added dropwised for 15 min. After the mixture was stirred for another 1 h, The corresponding diamide precipitated immediately. The product was washed with HCl, dried, and recrystallized from EtOH to give the title compounds **6a**~**6v**.

**2-(2-(4-ethoxy-3-methoxyphenyl)acetamido)-3-methyl-N-(1-phenylethyl)butanamide 6a**：white solid, yield 50%, ^1^H NMR (400 MHz, CDCl_3_): δ 7.39-7.25 (m, 5H, Ar-H), 6.87-6.73 (m, 3H, Ar-H), 6.21-5.67 (m, 2H, 2NH), 5.12-5.03 (m, 1H, NHCHCH_3_), 4.18-4.11 (m, 3H, (CH_3_)_2_CHCH, CH_3_CH_2_O), 3.85 (s, 3H, CH_3_O), 3.56-3.52 (m, 2H, CH_2_CO), 2.08-1.97 (m, 1H, (CH_3_)_2_CHCH), 1.49 (s, 6H, NHCHCH_3_, CH_3_CH_2_O), 0.94-0.76 (m, 6H, (CH_3_)_2_CH). HRMS (ESI) m/z Calcd for C_24_H_32_N_2_O_4_Na^+^[M+Na]^+^ 435.2254, Found: 435.2256.IR(KBr,cm^-1^): 700.16, 758.02, 792.74, 1037.7, 1159.22,1230.58, 1263.37, 1382.96, 1450.47, 1631.78, 2972.31, 3294.42.

**2-(2-(3-methoxy-4-propoxyphenyl)acetamido)-3-methyl-N-(1-phenylethyl)butanamide 6b**：white solid, yield 69%，^1^H NMR (400 MHz, CDCl_3_): δ 7.36-7.25 (m, 5H, Ar-H), 6.86-6.73 (m, 3H, Ar-H), 6.54 (s, 1H, NH), 6.22 (d, *J =* 22.8Hz, 1H, NH), 5.09-5.02 (m, 1H, NHCHCH_3_), 4.22 (t, *J =* 7.6Hz, 1H, (CH_3_)_2_CHCH), 3.99-3.95 (m, 2H, CH_3_CH_2_CH_2_O), 3.83 (d, *J =* 21.6Hz, 3H, OCH_3_), 3.58-3.50 (m, 2H, CH_2_OC), 2.06-1.95 (m, 1H, (CH_3_)_2_CHCH), 1.93-1.84 (m, 2H, CH_3_CH_2_CH_2_O), 1.46 (dd, *J*_1_ = 6.8Hz, *J*_2_ = 10.8Hz, 3H, NHCHCH_3_), 1.05 (t, *J =* 7.2Hz, 3H, CH_3_CH_2_CH_2_O), 0.93-0.75 (m, 6H, (CH_3_)_2_CH). HRMS (ESI) m/z Calcd for C_25_H_34_N_2_O_4_Na^+^[M+Na]^+^ 449.2411, Found: 449.2415. IR(KBr,cm^-1^): 700.16, 758.02, 786.96, 1039.63, 1161.15, 1230.58, 1263.37, 1631.78, 2964.59, 3294.42.

**2-(2-(3-methoxy-4-propoxyphenyl)acetamido)-3-methyl-N-(1-(p-tolyl)ethyl)butanamide 6c**：white solid, yield 64%，^1^H NMR (400 MHz, CDCl_3_): δ 7.20-7.12 (m, 4H, Ar-H), 6.86-6.73 (m, 3H, Ar-H), 6.45 (s, 1H, NH), 6.21 (s, 1H, NH), 5.05-4.99 (m, 1H, NHCHCH_3_), 4.22 (t, *J =* 7.2Hz, 1H, (CH_3_)_2_CHCH), 3.97 (t, *J =* 6.8Hz, 2H, CH_3_CH_2_CH_2_O), 3.83 (d, *J =* 20.0Hz, 3H, OCH_3_), 3.58-3.46 (m, 2H, CH_2_OC), 2.34 (s, 3H, p-CH_3_Ar), 2.05-1.94 (m, 1H, (CH_3_)_2_CHCH), 1.93-1.84 (m, 2H, CH_3_CH_2_CH_2_O), 1.44 (dd, *J*_1_ = 7.2Hz, *J*_2_ = 10.0Hz, 3H, NHCHCH_3_), 1.05 (t, *J =* 7.6Hz, 3H, CH_3_CH_2_CH_2_O), 0.92-0.75 (m, 6H, (CH_3_)_2_CH). HRMS (ESI) m/z Calcd for C_26_H_36_N_2_O_4_Na^+^[M+Na]^+^ 463.2567, Found: 463.2564. IR(KBr,cm^-1^): 698.23, 756.1, 785.03, 854.47, 1159.22, 1230.58, 1382.96, 1514.12, 1633.71, 2962.66, 3078.39, 3286.7.

**2-(2-(4-(allyloxy)-3-methoxyphenyl)acetamido)-3-methyl-N-(1-phenylethyl)butanamide 6d**：light red solid, yield 68%，^1^H NMR (400 MHz, CDCl_3_): δ 7.36-7.25 (m, 5H, Ar-H), 6.86-6.71 (m, 3H, Ar-H), 6.51 (s, 1H, NH), 6.20 (d, *J =* 24.0Hz, 1H, NH), 6.14-6.05 (m, 1H, CH_2_=CHCH_2_O), 5.36 (dd, *J*_1_ = 15.6Hz, *J*_2_=48.0Hz, 2H, CH_2_=CHCH_2_O), 5.08-5.03 (m, 1H, NHCHCH_3_), 4.61 (s, 2H, CH_2_=CHCH_2_O), 4.22 (t, *J =* 8.0Hz, 1H, (CH_3_)_2_CHCH), 3.84 (t, *J =* 22.0Hz, 3H, OCH_3_), 3.58-3.46 (m, 2H, CH_2_OC), 2.07-1.93 (m, 1H, (CH_3_)_2_CHCH), 1.46 (dd, *J*_1_ = 6.8Hz, *J*_2_=10.8Hz, 3H, NHCHCH_3_), 0.93-0.75 (m, 6H, (CH_3_)_2_CH). HRMS (ESI) m/z Calcd for C_25_H_32_N_2_O_4_Na^+^[M+Na]^+^ 447.2260, Found: 447.2252. IR(KBr,cm^-1^): 698.23, 785.03, 920.05, 1018.41, 1230.58, 1263.37, 1382.96, 1514.12, 1541.12, 1633.71, 2962.66, 3064.89, 3078.39, 3286.7.

**2-(2-(4-(allyloxy)-3-methoxyphenyl)acetamido)-3-methyl-N-(1-(p-tolyl)ethyl)butanamide 6e**：light red solid, yield 65%，^1^H NMR (400 MHz, CDCl_3_): δ 7.20-7.15 (m, 4H, Ar-H), 6.87-6.73 (m, 3H, Ar-H), 6.39 (s, 1H, NH), 6.21 (d, *J =* 21.2Hz, 1H, NH), 6.14-6.06 (m, 1H, CH_2_=CHCH_2_O), 5.36 (dd, *J*_1_ = 17.2Hz, *J*_2_ = 49.2Hz, 2H, CH_2_=CHCH_2_O), 5.04-5.00 (m, 1H, NHCHCH_3_), 4.61 (s, 2H, CH_2_=CHCH_2_O), 4.20 (t, *J =* 5.6Hz, 1H, (CH_3_)_2_CHCH), 3.85 (d, *J =* 20.4Hz, 3H, OCH_3_), 3.53 (d, *J =* 14Hz, 2H, CH_2_OC), 2.34 (s, 3H, p-CH_3_Ar), 2.07-1.99 (m, 1H, (CH_3_)_2_CHCH), 1.46 (t, *J =* 7.6Hz, 3H, NHCHCH_3_), 0.93-0.76 (m, 6H, (CH_3_)_2_CH). HRMS (ESI) m/z Calcd for C_26_H_34_N_2_O_4_H^+^[M+H]^+^ 439.2591, Found: 439.2598. IR(KBr,cm^-1^): 702.09, 786.96, 1018.41, 1159.22, 1230.58, 1263.37, 1381.03, 1516.05, 1541.12, 1631.78, 2960.73, 3059.1, 3282.84.

**2-(2-(3-methoxy-4-(prop-2-yn-1-yloxy)phenyl)acetamido)-3-methyl-N-(1-phenylethyl)butanamide 4f**：light yellow solid, yield 53%，^1^H NMR (400 MHz, CDCl_3_): δ 7.37-7.26 (m, 5H, Ar-H), 7.01 (t, *J =* 8.4Hz, 1H, Ar-H), 6.84-6.78 (m, 2H, Ar-H), 6.51 (s, 1H, NH), 6.26 (d, *J =* 15.6Hz, 1H, NH), 5.10-5.03 (m, 1H, NHCHCH_3_), 4.77 (s, 2H, OCH_2_CCH), 4.23 (t, *J =* 6.4Hz, 1H, (CH_3_)_2_CHCH), 3.85 (d, *J =* 23.6Hz, 3H, CH_3_O), 3.54 (d, *J =* 16.0Hz, 2H, CH_2_OC), 2.52 (s, 1H, OCH_2_CCH), 2.08-2.00 (m, 1H, (CH_3_)_2_CHCH), 1.48 (dd, *J*_1_ = 7.2Hz, *J*_2_ = 9.6Hz, 3H, NHCHCH_3_), 0.89 (dd, *J*_1_ = 6.4Hz, *J*_2_ = 32Hz, 3H, (CH_3_)_2_CH), 0.80 (dd, *J*_1_ = 6.4Hz, *J*_2_ = 14.8Hz, 3H, (CH_3_)_2_CH). HRMS (ESI) m/z Calcd for C_25_H_30_N_2_O_4_H^+^[M+H]^+^ 423.2278, Found: 423.2276. IR(KBr,cm^-1^): 696.3, 923.9, 1024.2, 1139.93, 1215.15, 1267.23, 1516.05, 1633.71, 2958.8, 3288.63.

**2-(2-(3-methoxy-4-(prop-2-yn-1-yloxy)phenyl)acetamido)-3-methyl-N-(1-(p-tolyl)ethyl)butanamide 6g**：light yellow solid, yield 83%，^1^H NMR (400 MHz, CDCl_3_): δ 7.19-7.15 (m, 4H, Ar-H), 7.00 (t, *J =* 7.6 Hz, 1H, Ar-H), 6.83-6.78 (m, 2H, Ar-H), 6.36 (s, 1H, NH), 6.22 (d, *J =* 20.0 Hz, 1H, NH), 5.08-5.00 (m, 1H, NHCHCH_3_), 4.77 (s, 2H, OCH_2_CCH), 4.02 (d, *J =* 6.0Hz, 1H, (CH_3_)_2_CHCH), 3.84 (d, *J =* 21.6Hz, 3H, CH_3_O), 3.54 (d, *J =* 13.6Hz, 2H, CH_2_OC), 2.51 (s, 1H, OCH_2_CCH), 2.34 (s, 3H, p-CH_3_Ar), 2.10-1.97 (m, 1H, (CH_3_)_2_CHCH), 1.46 (t, *J =* 8.0Hz, 3H, NHCHCH_3_), 0.93-0.77 (m, 3H, (CH_3_)_2_CH). HRMS (ESI) m/z Calcd for C_26_H_32_N_2_O_4_H^+^[M+H]^+^ 437.2435, Found: 437.2436. IR(KBr,cm^-1^): 785.03, 852.54, 921.97, 1024.2, 1139.93, 1215.15, 1267.23, 1514.12, 1543.05, 1631.78, 2960.73, 3059.1, 3275.13.

**3-methyl-N-(1-phenylethyl)-2-(2-(4-(prop-2-yn-1-yloxy)phenyl)acetamido)butanamide 6h**：yellow solid, yield 67%，^1^H NMR (400 MHz, CDCl_3_): δ 7.36-7.25 (m, 5H, Ar-H), 7.20 (d, *J =* 8.0Hz, 2H, Ar-H), 6.95 (d, *J =* 8.4Hz, 2H, Ar-H), 6.64 (s, 1H, NH), 6.32 (s, 1H, NH), 5.07-5.04 (m, 1H, NHCHCH_3_), 4.69 (s, 2H, OCH_2_CCH), 4.24 (t, *J =* 6.0Hz, 1H, (CH_3_)_2_CHCH), 3.54 (s, 2H, CH_2_OC), 2.53 (s, 1H, OCH_2_CCH), 2.07-1.91 (m, 1H, (CH_3_)_2_CHCH), 1.43 (d, *J =* 6.8Hz, 3H, NHCHCH_3_), 0.92-0.75 (m, 6H, (CH_3_)_2_CH). HRMS (ESI) m/z Calcd for C_24_H_28_N_2_O_3_H^+^[M+H]^+^ 393.2173, Found: 393.2175. IR(KBr,cm^-1^): 694.37, 754.17, 925.83, 1031.92, 1176.58, 1220.94, 1300.02, 1512.19, 1633.71, 2870.08, 2956.87, 3064.89, 3271.27.

**3-methyl-2-(2-(4-(prop-2-yn-1-yloxy)phenyl)acetamido)-N-(1-(p-tolyl)ethyl)butanamide 6i**：yellow solid, yield 50%，^1^H NMR (400 MHz, CDCl_3_): δ 7.22-7.15 (m, 6H, Ar-H), 6.96 (t, *J =* 8.4 Hz, 2H, Ar-H), 6.24 (dd, *J*_1_ = 8.4Hz, *J*_2_=17.2Hz, 1H, NH), 6.07 (dd, *J*_1_ = 8.0Hz, *J*_2_=20.8Hz, 1H, NH), 5.06-4.99 (m, 1H, NHCHCH_3_), 4.70 (s, 2H, OCH_2_CCH), 4.17 (t, *J =* 7.6Hz, 1H, (CH_3_)_2_CHCH), 3.55 (s, 2H, CH_2_OC), 2.54 (s, 1H, OCH_2_CCH), 2.35 (d, *J =* 4.4Hz, 3H, p-CH_3_Ar), 2.06-1.94 (m, 1H, (CH_3_)_2_CHCH), 1.45 (t, *J =* 9.2Hz, 3H, NHCHCH_3_), 0.92-0.76 (m, 6H, (CH_3_)_2_CH). HRMS (ESI) m/z Calcd for C_25_H_30_N_2_O_3_H^+^[M+H]^+^ 407.2329, Found: 407.2324. IR(KBr,cm^-1^): 817.82, 923.9, 1029.99,1103.28, 1219.01, 1301.95, 1512.19, 1544.98, 1633.71, 2115.91, 3064.89, 3273.2.

**3-methyl-2-(2-phenylacetamido)-N-(1-phenylethyl)butanamide 6j**：white solid, yield 59%，^1^H NMR (400 MHz, CDCl_3_): δ 7.32-7.24 (m, 10H, Ar-H), 6.53 (s, 1H, NH), 6.19 (dd, *J*_1_ = 8.0Hz, *J*_2_=19.6Hz ,1H, NH), 5.04 (t, *J =* 5.2Hz, 1H, (CH_3_)_2_CHCH), 4.21 (t, *J =* 8.0Hz, 1H, CH_3_CH), 3.56 (d, *J =* 16.0Hz, 2H, CH_2_ ), 2.03-1.90 (m, 1H, (CH_3_)_2_CH), 1.43 (dd, *J*_1_ = 6.8Hz, *J*_2_=14Hz ,1H, CH_3_CH), 0.89-0.72 (m, 6H, (CH_3_)_2_CH). HRMS (ESI) m/z Calcd for C_21_H_26_N_2_O_2_Na^+^[M+Na]^+^ 361.1886, Found: 361.1887. IR(KBr,cm^-1^): 696.3, 759.95, 1018.41, 1234.44, 1556.55, 1633.71, 2962.66, 3064.89, 3282.84.

**3-methyl-2-(2-phenylacetamido)-N-(1-(p-tolyl)ethyl)butanamide 6k**：white solid, yield 60%，^1^H NMR (400 MHz, CDCl_3_): δ 7.35-7.22 (m, 5H, Ar-H), 7.19-7.10 (m, 4H, Ar-H), 6.73 (s, 1H, NH), 6.35 (s, 1H, NH), 5.05-4.98 (m, 1H, NHCHCH_3_), 4.24 (t, *J =* 7.6Hz, 1H, (CH_3_)_2_CHCH), 3.54 (s, 2H, CH_2_OC), 2.34 (s, 3H, p-CH_3_-Ar), 2.04-1.91 (m, 1H, (CH_3_)_2_CHCH), 1.44 (d, *J =* 6.4Hz, 3H, NHCHCH_3_), 0.88 (d, *J =* 6.4Hz, 3H, (CH_3_)_2_CH), 0.81(d, *J =* 6.4Hz, 3H, (CH_3_)_2_CH). HRMS (ESI) m/z Calcd for C_22_H_28_N_2_O_2_Na^+^[M+Na]^+^ 375.2043, Found: 375.2041. IR(KBr,cm^-1^): 696.3, 813.96, 1018.41, 1238.3, 1548.84, 1633.71, 1676.14, 2970.38, 3280.92.

**2-(2-(4-chlorophenyl)acetamido)-N-(4-methoxybenzyl)-3-methylbutanamide 6l**：white solid, yield 67%，^1^H NMR (400 MHz, CDCl_3_): δ 7.29 (d, *J =* 8.0Hz, 2H, p-Cl-Ar-H), 7.16 (d, *J =* 8.4Hz, 2H, p-Cl-Ar-H), 7.14 (d, *J =* 8.4Hz, 2H, p-OMe-Ar-H), 6.83 (d, *J =* 8.4Hz, 2H, p-OMe-Ar-H), 6.39 (s, 1H, NH), 6.29 (s, 1H, NH), 4.39-4.21 (m, 2H, NHCH_2_), 3.79 (s, 3H, OCH_3_), 3.51 (s, 2H, COCH_2_), 2.01-2.00 (m, 1H, (CH_3_)_2_CHCH), 0.85 (dd, *J*_1_ = 6.0Hz, *J*_2_ = 18.4Hz, 6H, (CH_3_)_2_CH). HRMS (ESI) m/z Calcd for C_21_H_25_ClN_2_O_3_Na^+^[M+Na]^+^ 411.1446, Found: 411.1444. IR(KBr,cm^-1^): 713.66, 806.25, 1029.99, 1253.73, 1631.78, 1641.42, 1888.31.

**N-(2-methoxybenzyl)-3-methyl-2-(2-phenylacetamido)butanamide 6m**

^1^H NMR (400 MHz, CDCl_3_): δ 7.35-7.20 (m, 7H, Ar-H), 6.91-6.85 (m, 2H, Ar-H), 6.39 (s, 1H, NH), 6.19 (s, 1H, NH), 4.47-4.33 (m, 2H, NHCH_2_), 4.19 (s, 1H, (CH_3_)_2_CHCH), 3.83 (s, 3H, OCH_3_), 3.57 (s, 2H, COCH_2_), 2.00-1.98 (m, 1H, (CH_3_)_2_CHCH ), 0.79 (dd, *J*_1_ = 6.4 Hz, *J*_2_ = 15.6Hz, 6H, (CH_3_)_2_CH). HRMS (ESI) m/z Calcd for C_21_H_26_N_2_O_3_Na^+^[M+Na]^+^ 377.1836, Found: 377.1832. IR(KBr,cm^-1^): 756.1, 785.03, 1028.06, 1251.8, 1539.2, 1627.92, 2960.73, 3290.56.

**N-(2-chlorobenzyl)-3-methyl-2-(2-phenylacetamido)butanamide 6n**：white solid, yield 55%，^1^H NMR (400 MHz, CDCl_3_): δ 7.35-7.20 (m, 9H, Ar-H), 6.52 (s, 1H, NH), 6.06 (d, *J =* 6.8Hz, 1H, NH), 4.54-4.41 (m, 2H, NHCH_2_), 4.23 (t, *J =* 15.6Hz, 1H, (CH_3_)_2_CHCH), 3.57 (d, *J =* 2.8Hz, 2H, COCH_2_), 2.06-1.97 (m, 1H, CH_3_)_2_CHCH ), 0.80 (dd, *J*_1_ = 6.8Hz, *J*_2_ = 28.4Hz, 6H, (CH_3_)_2_CH). HRMS (ESI) m/z Calcd for C_20_H_23_ClN_2_O_2_Na^+^[M+Na]^+^ 381.1340, Found: 381.1345. IR(KBr,cm^-1^): 692.44, 744.52, 1039.63, 1244.09, 1541.12, 1637.56, 1681.93, 2960.73, 3064.89, 3080.32, 3273.2.

**2-(2-(4-chlorophenyl)acetamido)-3-methyl-N-(4-methylbenzyl)butanamide 6o**：white solid, yield 64%，^1^H NMR (400 MHz, CDCl_3_): δ 7.29 (d, *J =* 8.0 Hz, 2H, p-Cl-Ar-H), 7.17 (d, *J =* 8.0 Hz, 2H, p-Cl-Ar-H), 7.11 (s, 4H, p-Me-Ar-H), 6.30 (s, 1H, NH), 6.23 (s, 1H, NH), 4.43-4.29 (m, 2H, NHCH_2_), 4.20 (s, 1H, (CH_3_)_2_CHCH), 3.52 (s, 2H, COCH_2_), 2.33(s, 3H, CH_3_), 2.05-1.98 (m, 1H, (CH_3_)_2_CHCH), 0.85 (dd, *J*_1_ = 5.6Hz, *J*_2_ = 20.0Hz, 6H, (CH_3_)_2_CH). HRMS (ESI) m/z Calcd for C_21_H_25_ClN_2_O_2_Na^+^[M+Na]^+^ 395.1497, Found: 395.1494. IR(KBr,cm^-1^): 804.32, 854.47, 1014.56, 1089.78, 1234.44, 1492.9, 1629.85, 1639.49, 1896.03,2958.80,3049,46,3277.06.

**2-(2-(4-chlorophenyl)acetamido)-N-(2-methoxybenzyl)-3-methylbutanamide 6p**：white solid, yield 61%，^1^H NMR (400 MHz, CDCl_3_): δ 7.29-7.17 (m, 6H, Ar-H), 6.88 (dd, *J*_1_ = 7.6Hz, *J*_2_=16.4Hz, 2H, p-Me-Ar-H), 6.52 (s, 1H, NH), 6.43 (s, 1H, NH), 4.48-4.33 (m, 2H, NHCH_2_), 4.20 (s, 1H, (CH_3_)_2_CHCH), 3.83 (s, 3H, OCH_3_), 3.52 (s, 2H, COCH_2_), 2.01-1.98 (m, 1H, (CH_3_)_2_CHCH), 0.82 (dd, *J*_1_ = 3.6Hz, *J*_2_ = 6.0Hz, 6H, (CH_3_)_2_CH). HRMS (ESI) m/z Calcd for C_21_H_25_ClN_2_O_3_Na^+^[M+Na]^+^ 411.1446, Found: 411.1442. IR(KBr,cm^-1^): 746.45, 808.17, 858.32, 1016.49, 1089.78, 1244.09, 1490.97, 1543.05, 2960.73, 3068.75, 3084.18, 3277.06.

**2-(2-(4-chlorophenyl)acetamido)-3-methyl-N-(1-phenylethyl)butanamide 6q**：white solid, yield 60%，^1^H NMR (400 MHz, CDCl_3_): δ 7.32-7.13 (m, 9H, Ar-H), 6.28 (dd, *J*_1_ = 6.8Hz, *J*_2_=14.4Hz, 1H, NH), 6.18 (dd, *J*_1_ = 8.8Hz, *J*_2_ = 14.8Hz, 1H, NH), 5.08-5.01(m, 1H, NHCHCH_3_), 4.20-4.15 (m, 1H, (CH_3_)_2_CHCH), 3.51 (d, *J =* 17.2Hz, 2H, COCH_2_), 2.04-1.92 (m, 1H, (CH_3_)_2_CHCH), 1.45 (dd, *J*_1_ = 7.2Hz, *J*_2_ = 10.4Hz, 3H, NHCHCH_3_), 0.88 (dd, *J*_1_ = 6.8Hz, *J*_2_ = 26 Hz, 3H, (CH_3_)_2_CH), 0.77 (dd, *J*_1_ = 6.8Hz, *J*_2_ = 13.2Hz, 3H, (CH_3_)_2_CH). HRMS (ESI) m/z Calcd for C_21_H_25_ClN_2_O_3_Na^+^[M+Na]^+^ 395.1497, Found: 395.1494. IR(KBr,cm^-1^): 705.95, 765.74, 806.25, 1016.49, 1089.78, 1558.48, 1633.71, 2968.45, 3062.96, 3080.32, 3269.34.

**2-(2-(4-chlorophenyl)acetamido)-3-methyl-N-(1-(p-tolyl)ethyl)butanamide 6r**：white solid, yield 66%，^1^H NMR (400 MHz, CDCl_3_): δ 7.34-7.21 (m, 4H, Ar-H), 7.19-7.14 (m, 4H, Ar-H), 6.18-6.12 (m, 2H, 2NH), 5.03 (q, *J =* 6.8Hz, 1H, NHCHCH_3_), 4.17 (t, *J =* 7.2Hz, 1H, (CH_3_)_2_CHCH), 3.54 (d, *J =* 16.4Hz, 2H, CH_2_OC), 2.35 (d, *J =* 4.0Hz, 3H, p-CH_3_-Ar), 2.08-1.93 (m, 1H, (CH_3_)_2_CHCH), 1.46 (t, *J* = 7.2Hz, 3H, NHCHCH_3_), 0.94-0.78 (m, 6H, (CH_3_)_2_CH). HRMS (ESI) m/z Calcd for C_22_H_27_ClN_2_O_2_Na^+^[M+Na]^+^ 409.1653, Found: 409.1650. IR(KBr,cm^-1^): 808.17, 1016.49, 1091.71, 1236.37, 1635.64, 2968.45, 3064.89, 3084.18, 3278.99.

**(E)-2-(3-(3,4-dimethoxyphenyl)acrylamido)-3-methyl-N-(1-phenylethyl)butanamide 6s**：yellow solid, yield 50%，^1^H NMR (400 MHz, CDCl_3_): δ 7.61 (d, *J =* 15.2Hz, 1H, CH=CHOC), 7.40-7.32 (m, 6H, Ar-H, CH=CHOC), 7.13-7.05 (m, 2H, Ar-H), 6.92-6.84 (m, 1H, Ar-H), 6.48 (s, 1H, NH), 5.12 (s, 1H, NH), 4.60-4.51 (m, 1H, NHCHCH_3_), 3.95-3.82 (m, 7H, 2CH_3_O, (CH_3_)_2_CHCH), 2.31-2.16 (m, 1H, (CH_3_)_2_CHCH), 1.60 (d, *J =* 6.4Hz, 3H, NHCHCH_3_), 1.05-0.89 (m, 6H, (CH_3_)_2_CH). HRMS (ESI) m/z Calcd for C_24_H_30_N_2_O_4_Na^+^[M+Na]^+^ 433.2098, Found: 433.2093. IR(KBr,cm^-1^): 700.16, 748.38, 970.19, 1022.27, 1136.07, 1257.59, 1643.35, 2835.36, 2866.22, 2933.73, 2960.73, 3275.13.

**2-(3-(3,4-dimethoxyphenyl)propanamido)-3-methyl-N-(1-phenylethyl)butanamide 6t**：white solid, yield 64%，^1^H NMR (400 MHz, CDCl_3_): δ 7.33-7.30 (m, 5H, Ar-H), 6.80-6.72 (m, 3H, Ar-H), 6.66 (s, 1H, NH), 6.39 (s, 1H, NH), 5.08 (s, 1H, NHCHCH_3_), 4.26 (s, 1H, (CH_3_)_2_CHCH), 3.86 (s, 6H, OMe), 2.92-2.84 (m, 2H, CH_2_CH_2_OC), 2.56-2.51 (m, 2H, CH_2_CH_2_OC), 2.04-1.96 (m, 1H, (CH_3_)_2_CHCH), 1.50 (t, *J =* 7.2Hz, 3H, NHCHCH_3_), 0.93-0.73 (m, 6H, (CH_3_)_2_CH). HRMS (ESI) m/z Calcd for C_24_H_32_N_2_O_4_Na^+^[M+Na]^+^ 435.2254, Found: 435.2258. IR(KBr,cm^-1^): 698.23, 800.46, 1024.2, 1238.3, 1253.73, 1633.71, 2960.73, 3288.63.

**2-(3-(3-methoxy-4-(prop-2-yn-1-yloxy)phenyl)propanamido)-3-methyl-N-(1-phenylethyl)butanamide 6u**：yellow solid, yield 51%，^1^H NMR (400 MHz, CDCl_3_): δ 7.34-7.29 (m, 5H, Ar-H), 6.99-6.72 (m, 3H, Ar-H), 6.52 (s, 1H, NH), 5.01 (s, 1H, NH), 4.75 (s, 2H, OCH_2_CCH), 4.29-4.15 (m, 1H, NHCHCH_3_), 3.86 (s, 3H, CH_3_O), 3.14 (s, 1H, (CH_3_)_2_CHCH), 2.94-2.90 (m, 2H, ArCH_2_CH_2_OC), 2.65-2.54 (m, 2H, ArCH_2_CH_2_OC), 2.51 (s, 1H, OCH_2_CCH), 2.07-1.99 (m, 1H, (CH_3_)_2_CHCH), 1.48 (s, 3H, NHCHCH_3_), 0.94-0.80 (m, 6H, (CH_3_)_2_CH). HRMS (ESI) m/z Calcd for C_26_H_32_N_2_O_4_Na^+^[M+Na]^+^ 459.2254, Found: 459.2256. IR(KBr,cm^-1^): 698.23, 800.46, 850.61, 1024.2, 1213.23, 1257.59, 1633.71, 1737.86, 2115.91, 2962.66, 3062.96, 3284.77.

**2-(3-(3-methoxy-4-(prop-2-yn-1-yloxy)phenyl)propanamido)-3-methyl-N-(1-(p-tolyl)ethyl)butanamide 6v**：brown solid, yield 49%，^1^H NMR (400 MHz, CDCl_3_): δ 7.24-7.13 (m, 3H, Ar-H), 6.98-6.92 (m, 1H, Ar-H), 6.79-6.74 (m, 3H, Ar-H)，6.03-5.97 (s, 1H, NH), 5.07-5.04 (d, *J* = 20Hz, 1H, NH), 4.74 (s, 2H, OCH_2_CCH), 4.61-4.55 (m, 1H, NHCHCH_3_), 4.25 (s, 1H, (CH_3_)_2_CHCH), 3.87 (s, 3H, CH_3_O), 3.01-2.85 (m, 2H, ArCH_2_CH_2_OC), 2.64-2.57 (m, 2H, ArCH_2_CH_2_OC), 2.51 (s, 1H, OCH_2_CCH), 2.34 (s，1H, p-CH_3_-Ar), 2.20-2.08 (m, 1H, (CH_3_)_2_CHCH), 1.49 (s, 3H, NHCHCH_3_), 0.92-0.80 (m, 6H, (CH_3_)_2_CH). HRMS (ESI) m/z Calcd for C_27_H_34_N_2_O_4_Na^+^[M+Na]^+^ 473.2411, Found: 473.2403. IR(KBr,cm^-1^): 806.25, 1024.2, 1213.23, 1259.52, 1631.78, 1732.08, 2117.84, 2964.59, 3061.03, 3282.84.

**^1^H**-**NMR and HRMS of 6**


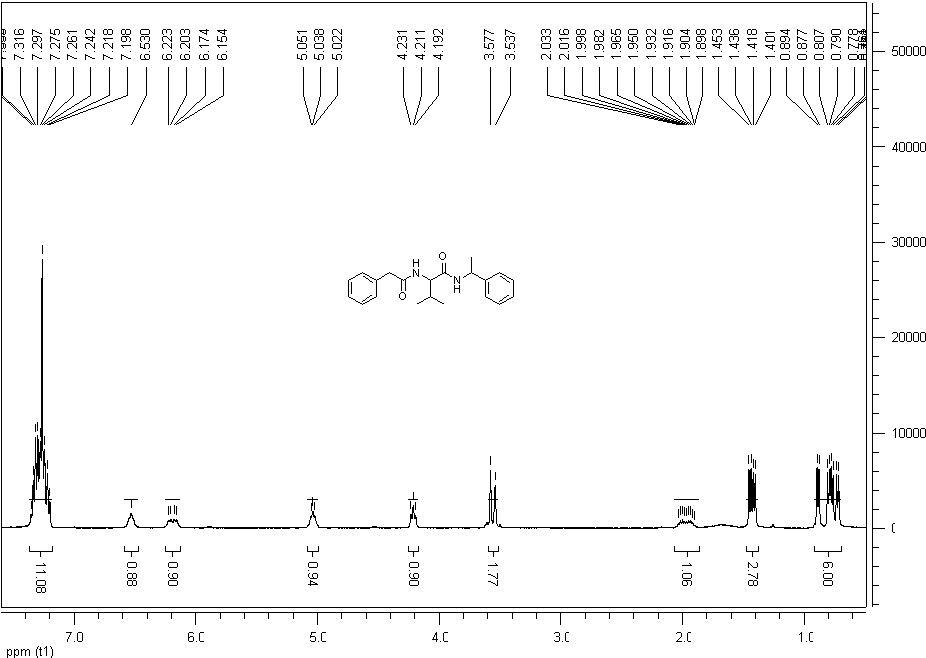


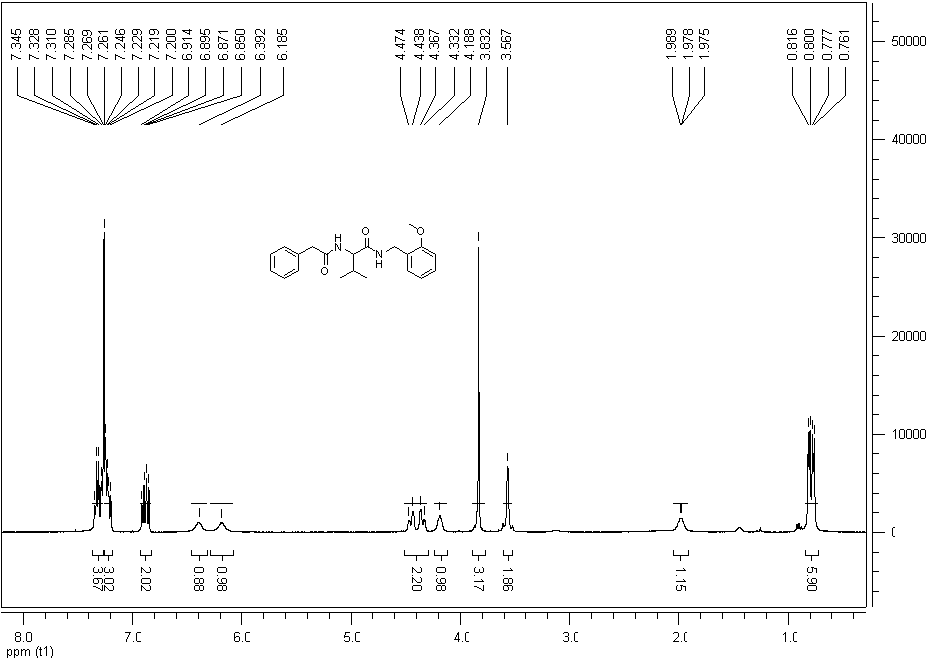


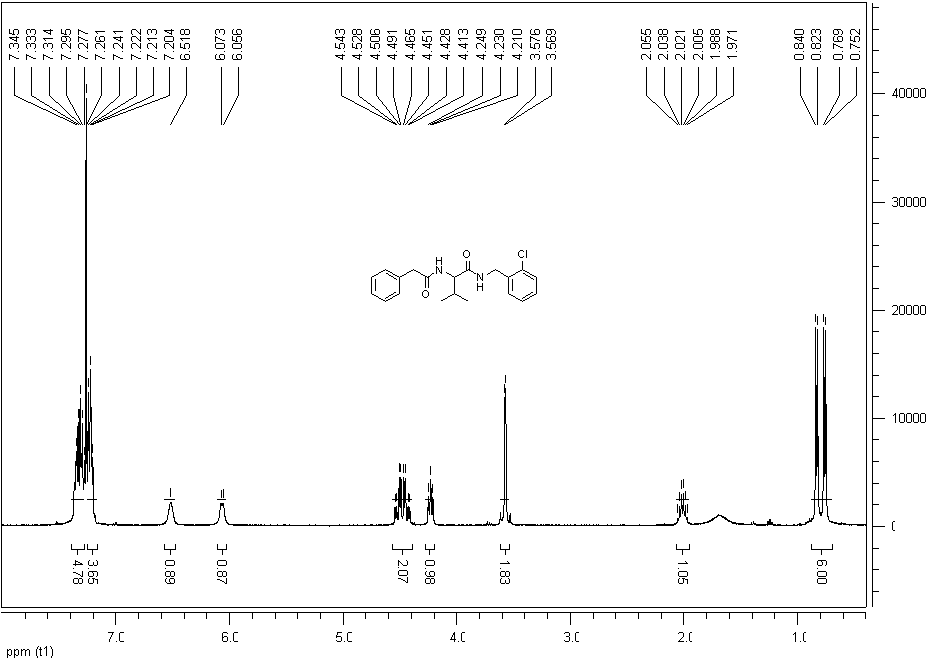


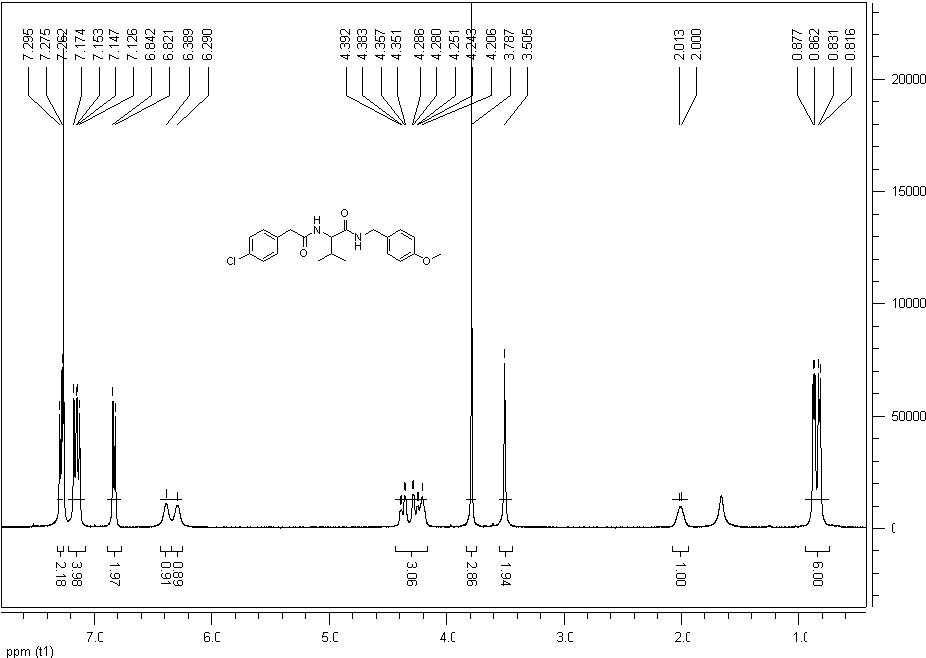


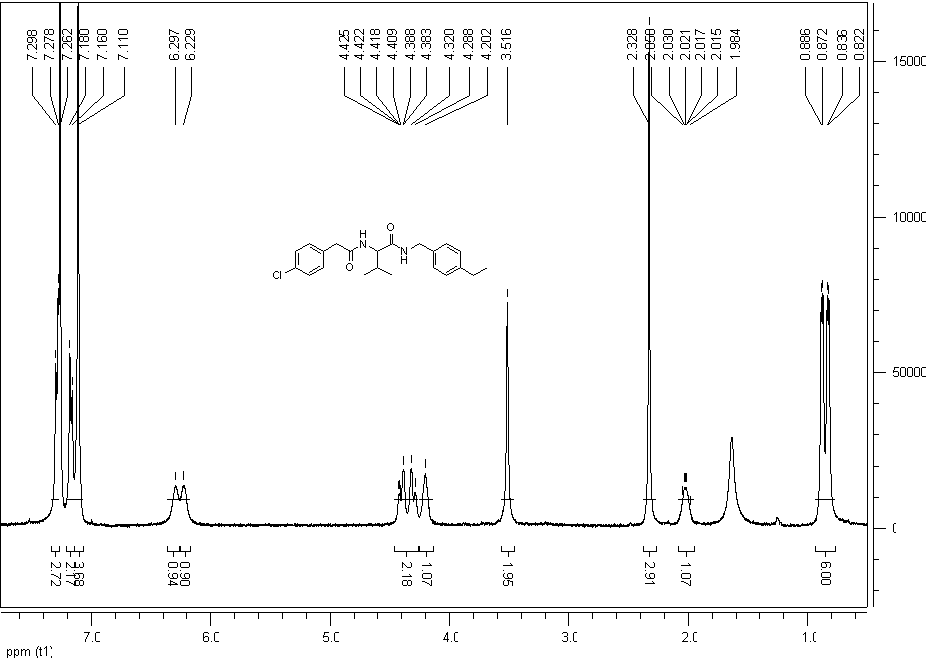


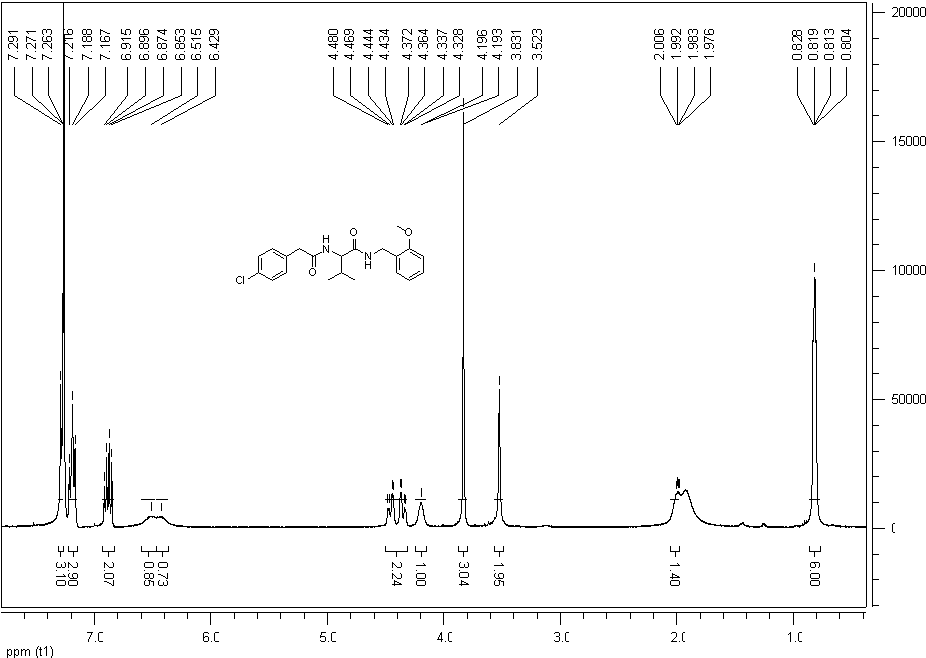


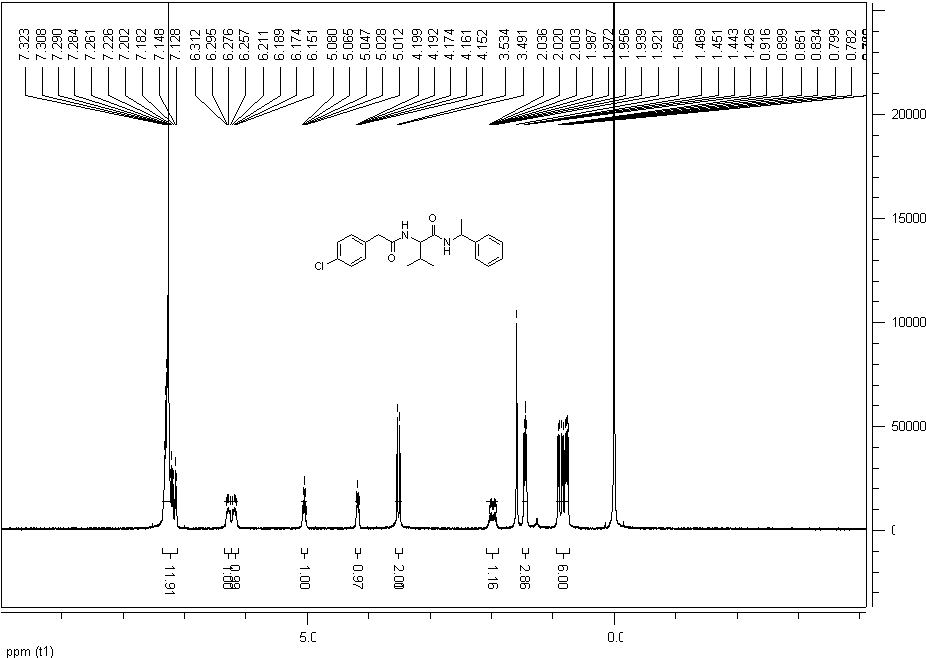


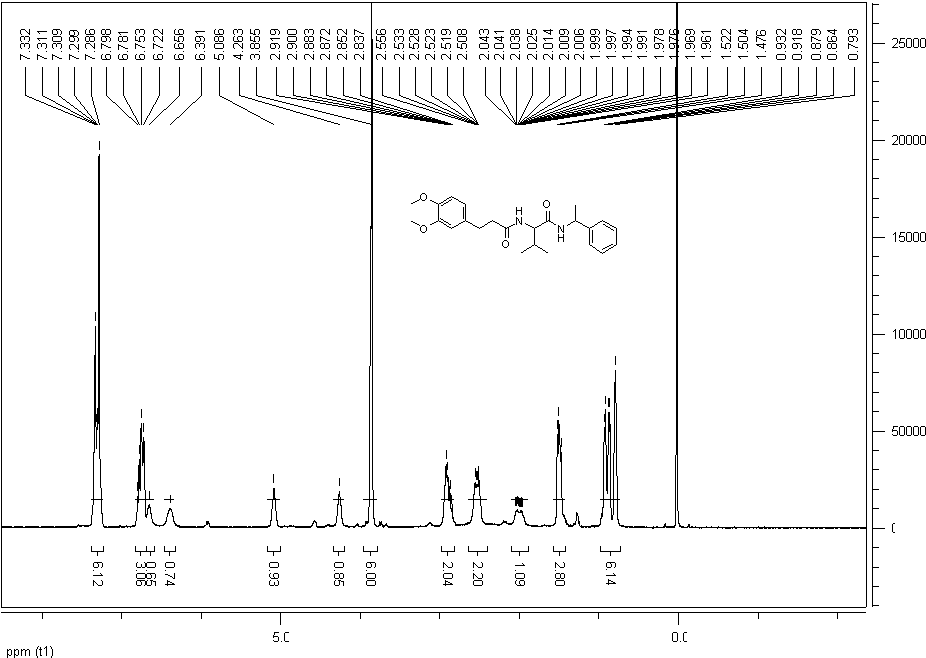


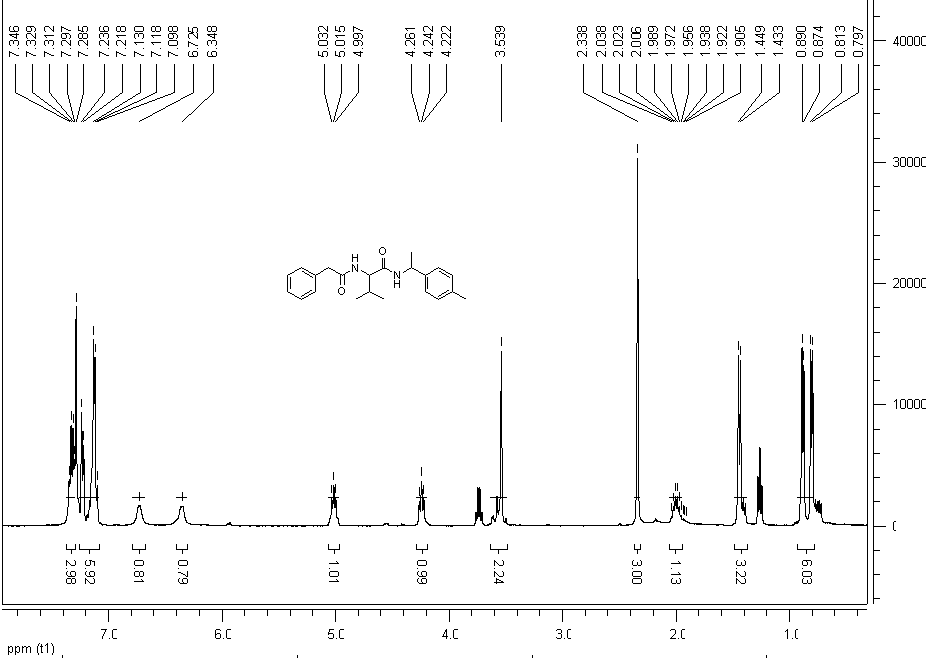


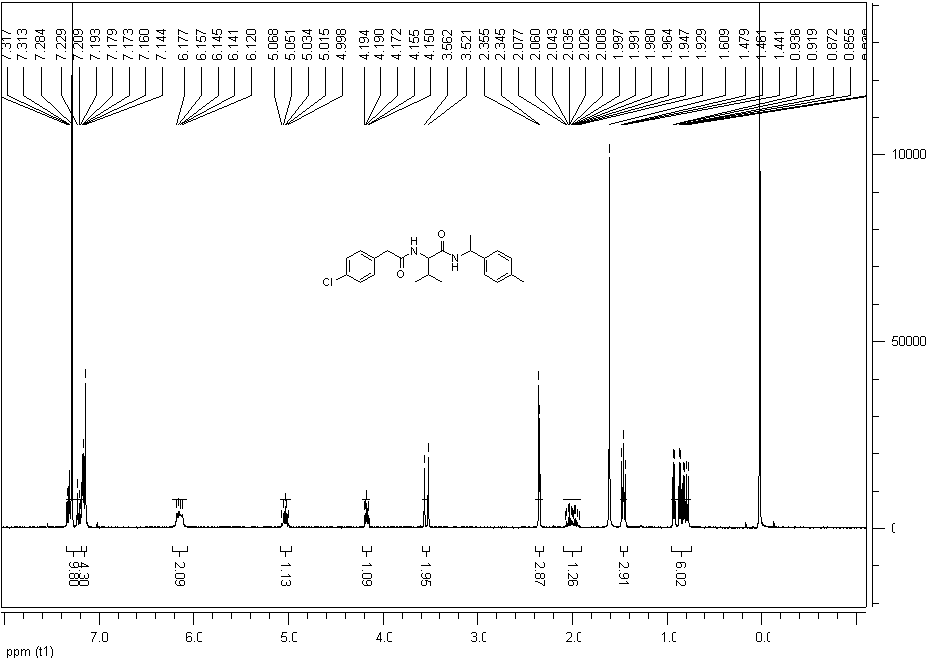


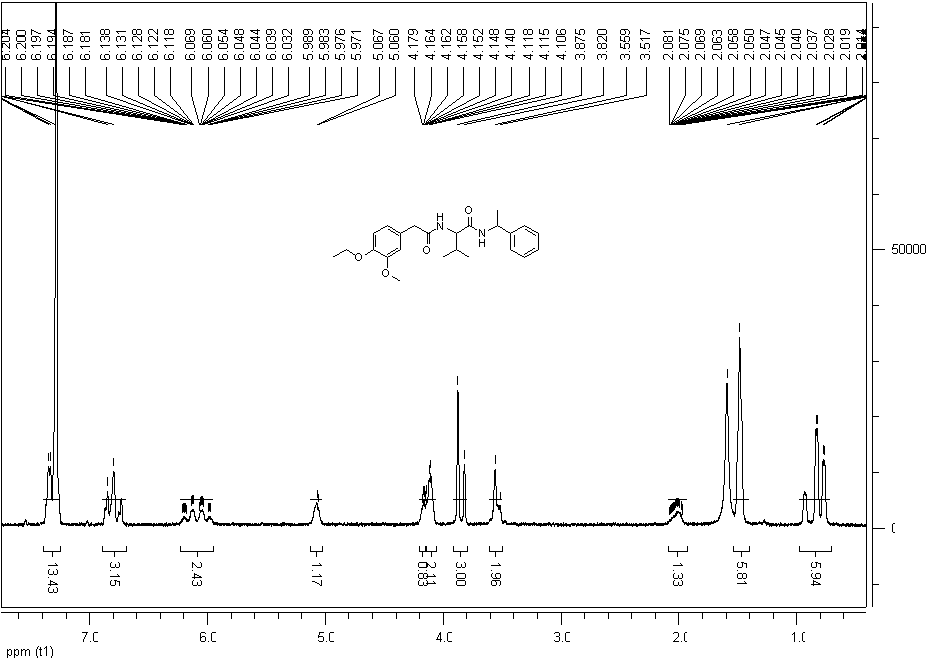


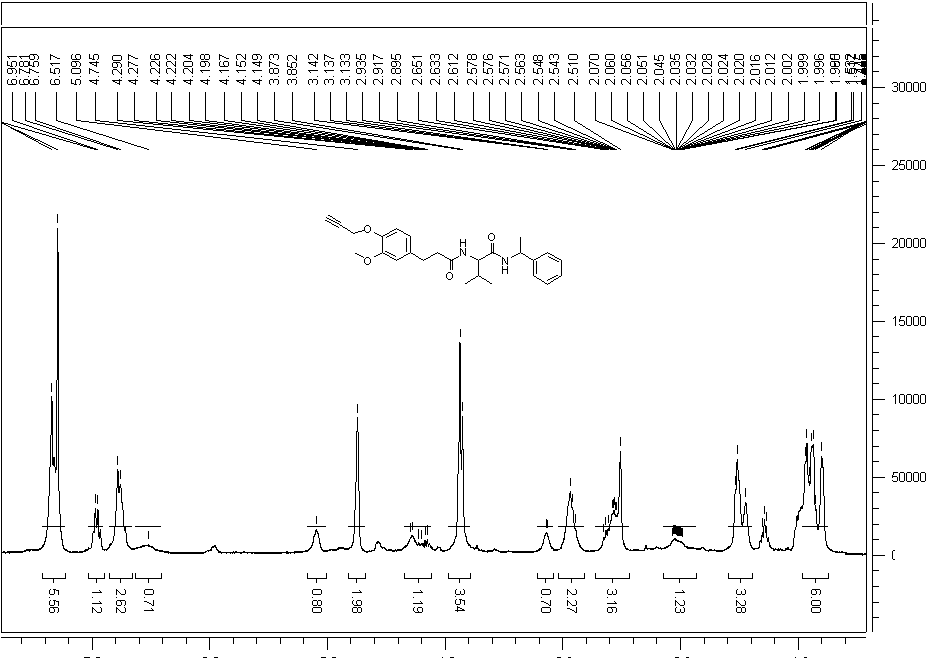


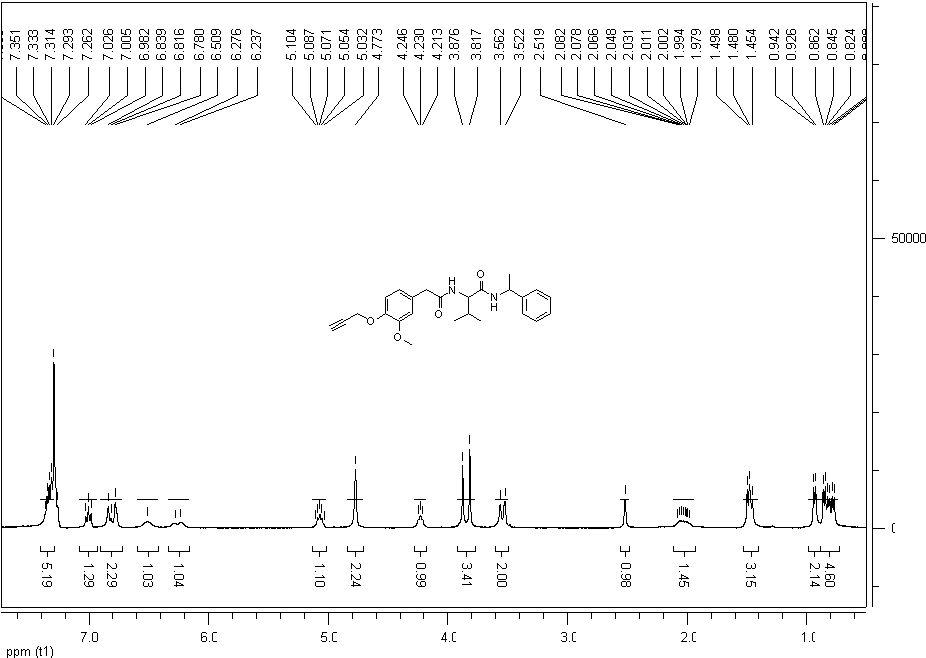


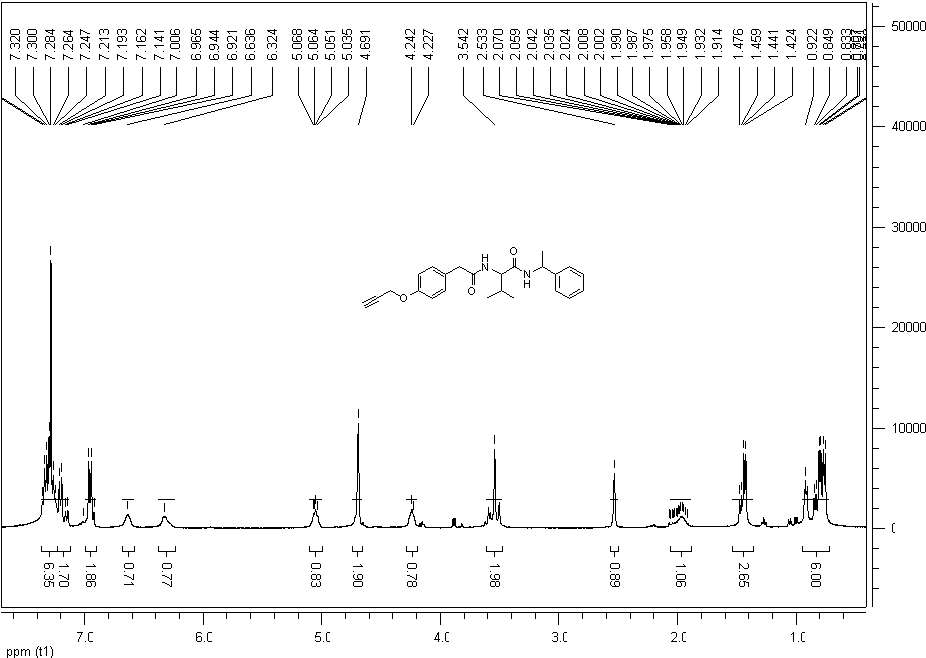


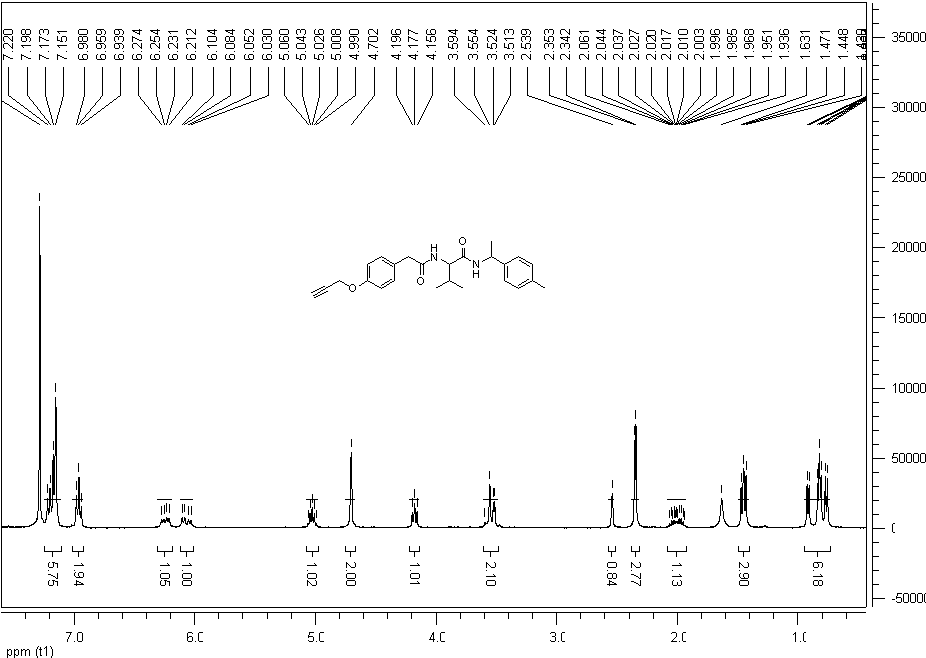


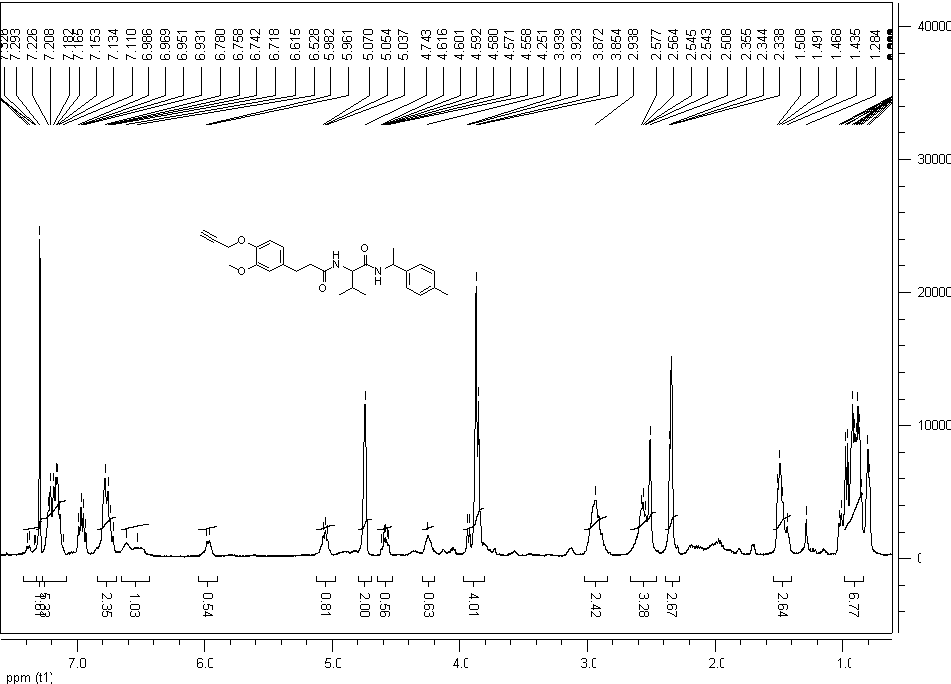


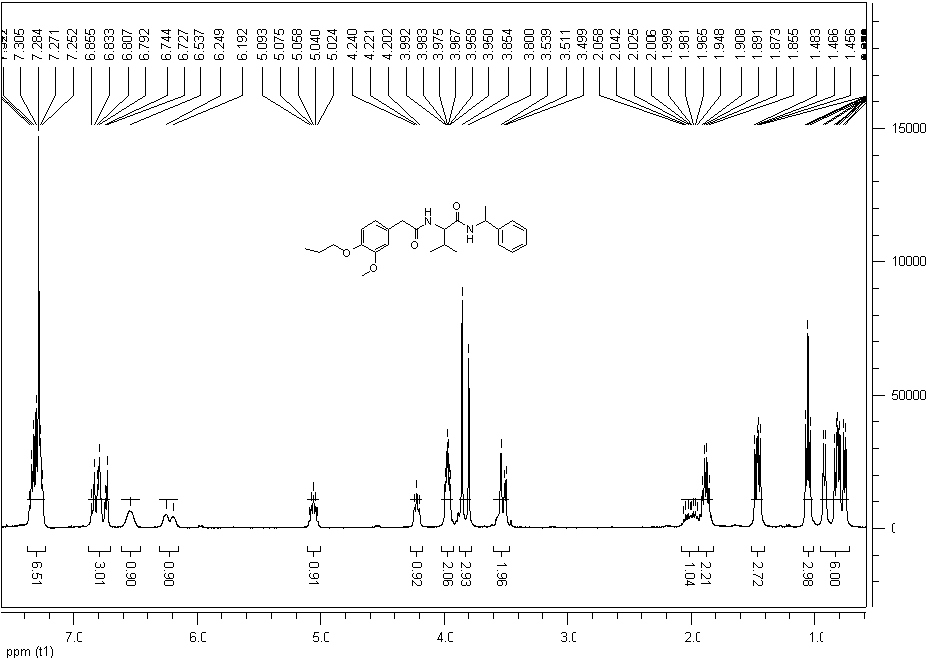


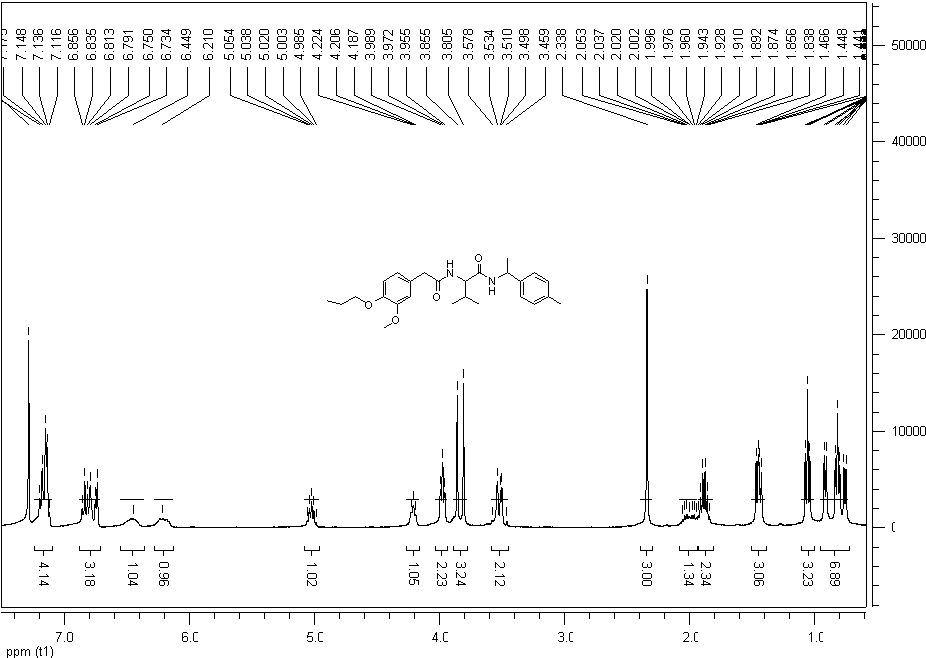


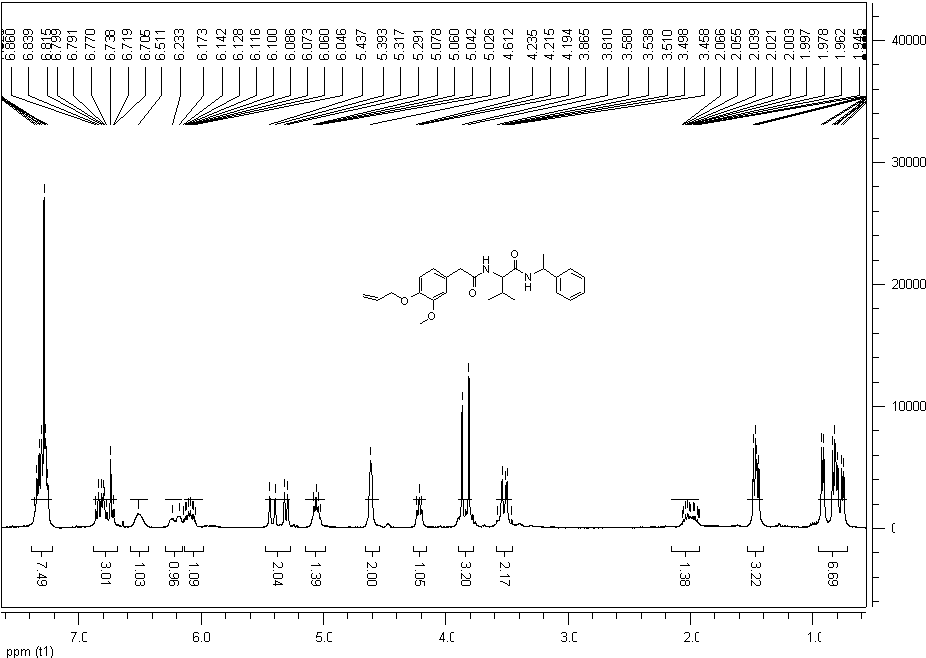


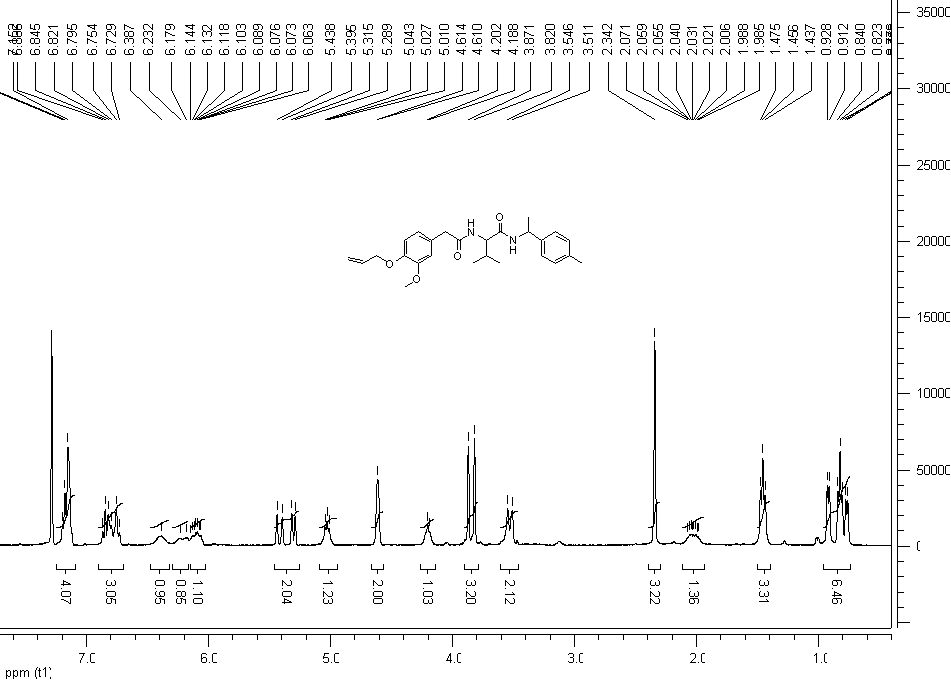


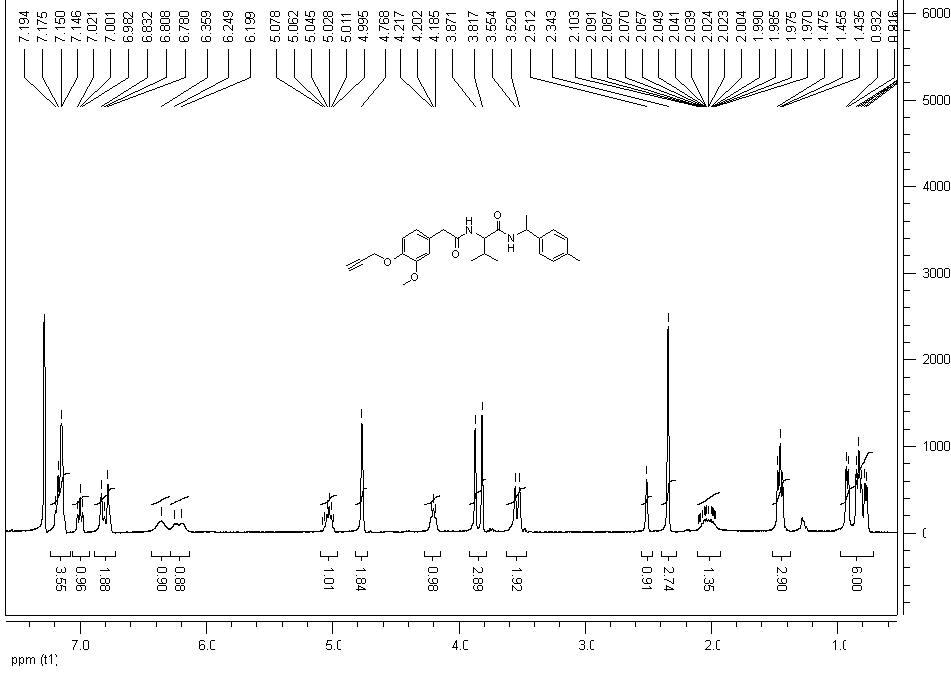


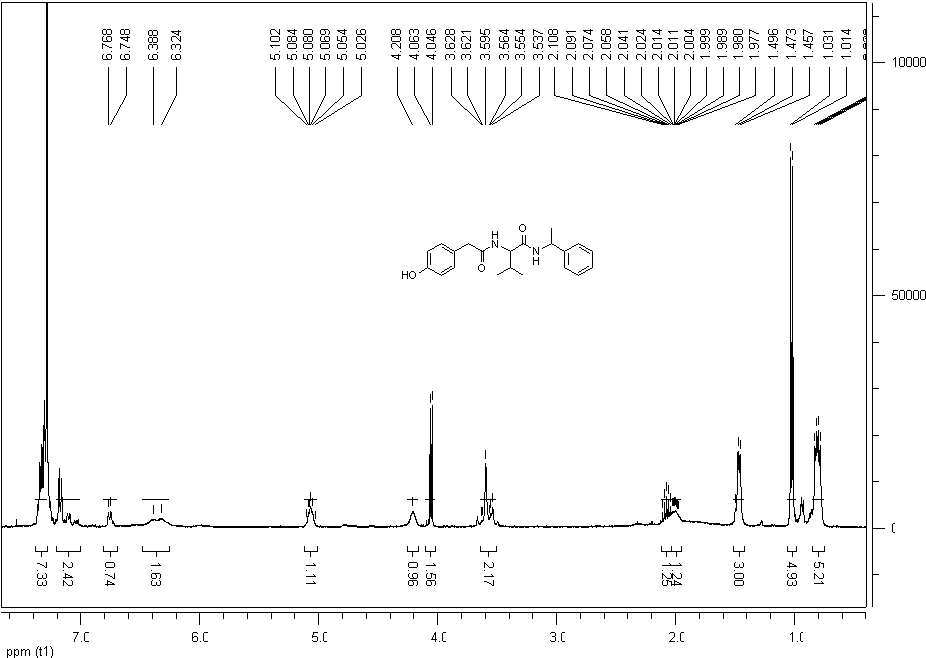

Supplement: Additional file 1 — Supporting information. [file 1752-153X-6-99-S1.doc]
