# Supplementary material for: Synthesis, crystal structure and larvicidal activity of novel diamide derivatives against Culex pipiens
Source: Chem Cent J. 2012 Sep 11;6:99. doi: 10.1186/1752-153X-6-99 (PMC3537643; doi:10.1186/1752-153X-6-99)
Supplement: Additional file 2 — Contains the CIF for compound 1. [file 1752-153X-6-99-S2.doc]

Additional file 3

**Synthesis, Crystal Structure and Larvicidal Activity of Novel Diamide Derivatives against *Culex pipiens***

Ru Wu^1^, Cong Zhu^1^, Xiu-Jiang Du^1^, Li-xia Xiong^1^, Shu-Jing Yu^1^, Xing-Hai Liu^2*^, Zheng-Ming Li^1^, Wei-Guang Zhao^1*^

Address: *1. State Key Laboratory of Elemento-Organic Chemistry, National Pesticide Engineering Research Center(Tianjin), Nankai University, Tianjin 300071, China*

*2. College of Chemical Engineering & Materials Sciences, Zhejiang University of Technology, Hangzhou, 310014, China*

Author to whom correspondence should be addressed;

Tel.: *Corresponding author Xing-Hai Liu. Tel: +86 571 88320147; Fax: +86 571 88320147; Wei-Guang Zhao. Tel: +86 22 23500074 Fax: +86 22 23500074;

E-Mail: WR: [wurui08@mail.nankai.edu.cn](mailto:wurui08@mail.nankai.edu.cn)

CZ: [zhucong@mail.nankai.edu.cn](mailto:zhucong@mail.nankai.edu.cn)

XJD: [dxj881216@126.com](mailto:dxj881216@126.com)

LXX: [xionglixia@nankai.edu.cn](mailto:xionglixia@nankai.edu.cn)

YSJ: [yushujing@nankai.edu.cn](mailto:yushujing@nankai.edu.cn)

XHL: [xhliu@zjut.edu.cn](mailto:xhliu@zjut.edu.cn)

ZML: [zml@nankai.edu.cn](mailto:zml@nankai.edu.cn)

WGZ: [zwg@nankai.edu.cn](mailto:zwg@nankai.edu.cn)

Additional data file 3 contains the CIF for compound 1

CCDC-891103 contains the supplementary crystallographic data for this paper. These data can be obtained free of charge at www.ccdc.cam.ac.uk/conts/retrieving.html or from the Cambridge Crystallographic Data Centre, 12 Union Road, Cambridge CB2 1EZ, UK; fax: +44-1223-336033; e-mail: [deposit@ccdc.cam.ac.uk](mailto:deposit@ccdc.cam.ac.uk).

Table 1. Crystal data and structure refinement for 6n.

Identification code 6n

Empirical formula C_20_ H_23_ Cl N_2_ O_2_

Formula weight 358.85

Temperature 293(2) K

Wavelength 0.71073 A

Crystal system, space group Triclinic, P-1

Unit cell dimensions a = 9.4583(19) A alpha = 70.49(3) deg.

b = 10.494(2) A beta = 73.27(3) deg.

c = 11.545(2) A gamma = 64.00(3) deg.

Volume 957.2(3) A^3

Z, Calculated density 2, 1.245 Mg/m^3

Absorption coefficient 0.214 mm^-1

F(000) 380

Crystal size 0.20 x 0.18 x 0.14 mm

Theta range for data collection 2.55 to 27.85 deg.

Limiting indices -12<=h<=12, -13<=k<=12, -15<=l<=14

Reflections collected / unique 9910 / 4491 [R(int) = 0.0405]

Completeness to theta = 27.85 98.5 %

Absorption correction Semi-empirical from equivalents

Max. and min. transmission 0.9706 and 0.9584

Refinement method Full-matrix least-squares on F^2

Data / restraints / parameters 4491 / 2 / 237

Goodness-of-fit on F^2 1.043

Final R indices [I>2sigma(I)] R1 = 0.0476, wR2 = 0.0971

R indices (all data) R1 = 0.1115, wR2 = 0.1174

Extinction coefficient 0.060(5)

Largest diff. peak and hole 0.307 and -0.339 e.A^-3

Table 2. Atomic coordinates ( x 10^4) and equivalent isotropic

displacement parameters (A^2 x 10^3) for 6n.

________________________________________________________________

x y z U(eq)

________________________________________________________________

Cl(1) 20(1) 7524(1) 10309(1) 82(1)

O(1) 1345(2) 5362(2) 3143(1) 55(1)

O(2) 3309(2) 5685(2) 6223(1) 58(1)

N(1) 3282(2) 4726(2) 4248(2) 41(1)

N(2) 957(2) 5479(2) 7195(2) 45(1)

C(1) 5831(3) 2602(3) 1921(2) 62(1)

C(2) 6306(4) 1325(3) 1564(2) 83(1)

C(3) 5424(4) 1251(4) 837(3) 91(1)

C(4) 4084(4) 2430(4) 476(2) 85(1)

C(5) 3638(3) 3700(3) 836(2) 62(1)

C(6) 4505(3) 3803(3) 1566(2) 45(1)

C(7) 3959(2) 5173(2) 2001(2) 46(1)

C(8) 2736(3) 5117(2) 3184(2) 40(1)

C(9) 2321(2) 4392(2) 5454(2) 41(1)

C(10) 2929(3) 2742(2) 6067(2) 56(1)

C(11) 4632(4) 2133(3) 6290(3) 93(1)

C(12) 2726(4) 1907(3) 5301(2) 90(1)

C(13) 2250(2) 5257(2) 6312(2) 40(1)

C(14) 750(3) 6123(2) 8198(2) 46(1)

C(15) -48(2) 7760(3) 7913(2) 46(1)

C(16) -476(3) 8604(3) 6764(2) 66(1)

C(17) -1182(4) 10105(3) 6523(3) 96(1)

C(18) -1478(4) 10801(3) 7430(3) 99(1)

C(19) -1089(3) 10002(3) 8581(3) 82(1)

C(20) -404(3) 8500(3) 8822(2) 56(1)

________________________________________________________________

Table 3. Bond lengths [A] and angles [deg] for shelxl.

_____________________________________________________________

Cl(1)-C(20) 1.737(2)

O(1)-C(8) 1.237(2)

O(2)-C(13) 1.233(2)

N(1)-C(8) 1.337(2)

N(1)-C(9) 1.453(2)

N(1)-H(1) 0.876(9)

N(2)-C(13) 1.334(2)

N(2)-C(14) 1.453(2)

N(2)-H(2) 0.893(9)

C(1)-C(6) 1.377(3)

C(1)-C(2) 1.379(3)

C(1)-H(1A) 0.9300

C(2)-C(3) 1.382(4)

C(2)-H(2A) 0.9300

C(3)-C(4) 1.377(4)

C(3)-H(3) 0.9300

C(4)-C(5) 1.379(3)

C(4)-H(4) 0.9300

C(5)-C(6) 1.389(3)

C(5)-H(5) 0.9300

C(6)-C(7) 1.507(3)

C(7)-C(8) 1.518(3)

C(7)-H(7A) 0.9700

C(7)-H(7B) 0.9700

C(9)-C(13) 1.523(2)

C(9)-C(10) 1.540(3)

C(9)-H(9) 0.9800

C(10)-C(11) 1.516(3)

C(10)-C(12) 1.532(3)

C(10)-H(10) 0.9800

C(11)-H(11A) 0.9600

C(11)-H(11B) 0.9600

C(11)-H(11C) 0.9600

C(12)-H(12A) 0.9600

C(12)-H(12B) 0.9600

C(12)-H(12C) 0.9600

C(14)-C(15) 1.503(3)

C(14)-H(14A) 0.9700

C(14)-H(14B) 0.9700

C(15)-C(16) 1.385(3)

C(15)-C(20) 1.389(3)

C(16)-C(17) 1.378(3)

C(16)-H(16) 0.9300

C(17)-C(18) 1.368(4)

C(17)-H(17) 0.9300

C(18)-C(19) 1.367(4)

C(18)-H(18) 0.9300

C(19)-C(20) 1.377(3)

C(19)-H(19) 0.9300

C(8)-N(1)-C(9) 121.66(17)

C(8)-N(1)-H(1) 117.8(15)

C(9)-N(1)-H(1) 120.2(15)

C(13)-N(2)-C(14) 122.77(16)

C(13)-N(2)-H(2) 116.3(12)

C(14)-N(2)-H(2) 120.8(12)

C(6)-C(1)-C(2) 121.5(2)

C(6)-C(1)-H(1A) 119.2

C(2)-C(1)-H(1A) 119.2

C(1)-C(2)-C(3) 119.1(3)

C(1)-C(2)-H(2A) 120.5

C(3)-C(2)-H(2A) 120.5

C(4)-C(3)-C(2) 120.7(3)

C(4)-C(3)-H(3) 119.7

C(2)-C(3)-H(3) 119.7

C(3)-C(4)-C(5) 119.2(3)

C(3)-C(4)-H(4) 120.4

C(5)-C(4)-H(4) 120.4

C(4)-C(5)-C(6) 121.2(3)

C(4)-C(5)-H(5) 119.4

C(6)-C(5)-H(5) 119.4

C(1)-C(6)-C(5) 118.3(2)

C(1)-C(6)-C(7) 121.1(2)

C(5)-C(6)-C(7) 120.6(2)

C(6)-C(7)-C(8) 109.34(15)

C(6)-C(7)-H(7A) 109.8

C(8)-C(7)-H(7A) 109.8

C(6)-C(7)-H(7B) 109.8

C(8)-C(7)-H(7B) 109.8

H(7A)-C(7)-H(7B) 108.3

O(1)-C(8)-N(1) 123.32(18)

O(1)-C(8)-C(7) 120.86(18)

N(1)-C(8)-C(7) 115.75(18)

N(1)-C(9)-C(13) 110.96(15)

N(1)-C(9)-C(10) 111.75(17)

C(13)-C(9)-C(10) 110.91(17)

N(1)-C(9)-H(9) 107.7

C(13)-C(9)-H(9) 107.7

C(10)-C(9)-H(9) 107.7

C(11)-C(10)-C(12) 111.6(2)

C(11)-C(10)-C(9) 112.71(19)

C(12)-C(10)-C(9) 110.5(2)

C(11)-C(10)-H(10) 107.3

C(12)-C(10)-H(10) 107.3

C(9)-C(10)-H(10) 107.3

C(10)-C(11)-H(11A) 109.5

C(10)-C(11)-H(11B) 109.5

H(11A)-C(11)-H(11B) 109.5

C(10)-C(11)-H(11C) 109.5

H(11A)-C(11)-H(11C) 109.5

H(11B)-C(11)-H(11C) 109.5

C(10)-C(12)-H(12A) 109.5

C(10)-C(12)-H(12B) 109.5

H(12A)-C(12)-H(12B) 109.5

C(10)-C(12)-H(12C) 109.5

H(12A)-C(12)-H(12C) 109.5

H(12B)-C(12)-H(12C) 109.5

O(2)-C(13)-N(2) 122.72(17)

O(2)-C(13)-C(9) 123.19(17)

N(2)-C(13)-C(9) 114.07(16)

N(2)-C(14)-C(15) 114.73(17)

N(2)-C(14)-H(14A) 108.6

C(15)-C(14)-H(14A) 108.6

N(2)-C(14)-H(14B) 108.6

C(15)-C(14)-H(14B) 108.6

H(14A)-C(14)-H(14B) 107.6

C(16)-C(15)-C(20) 116.7(2)

C(16)-C(15)-C(14) 122.99(19)

C(20)-C(15)-C(14) 120.3(2)

C(17)-C(16)-C(15) 121.7(2)

C(17)-C(16)-H(16) 119.1

C(15)-C(16)-H(16) 119.1

C(18)-C(17)-C(16) 120.0(3)

C(18)-C(17)-H(17) 120.0

C(16)-C(17)-H(17) 120.0

C(19)-C(18)-C(17) 119.9(3)

C(19)-C(18)-H(18) 120.1

C(17)-C(18)-H(18) 120.1

C(18)-C(19)-C(20) 119.9(2)

C(18)-C(19)-H(19) 120.1

C(20)-C(19)-H(19) 120.1

C(19)-C(20)-C(15) 121.8(2)

C(19)-C(20)-Cl(1) 118.39(19)

C(15)-C(20)-Cl(1) 119.8(2)

_____________________________________________________________

Symmetry transformations used to generate equivalent atoms:

Table 4. Anisotropic displacement parameters (A^2 x 10^3) for 6n.

_______________________________________________________________________

U11 U22 U33 U23 U13 U12

_______________________________________________________________________

Cl(1) 110(1) 94(1) 56(1) -38(1) -10(1) -38(1)

O(1) 37(1) 85(1) 52(1) -24(1) -5(1) -27(1)

O(2) 43(1) 95(1) 62(1) -44(1) 7(1) -41(1)

N(1) 33(1) 60(1) 39(1) -23(1) 1(1) -20(1)

N(2) 43(1) 62(1) 44(1) -30(1) 5(1) -29(1)

C(1) 60(2) 68(2) 64(2) -26(1) -2(1) -29(2)

C(2) 81(2) 74(2) 90(2) -31(2) 7(2) -30(2)

C(3) 98(3) 96(3) 103(2) -64(2) 28(2) -56(2)

C(4) 88(2) 128(3) 76(2) -62(2) 15(2) -65(2)

C(5) 63(2) 85(2) 51(1) -35(1) 0(1) -33(2)

C(6) 45(1) 60(2) 35(1) -18(1) 4(1) -28(1)

C(7) 50(1) 57(2) 40(1) -16(1) -2(1) -29(1)

C(8) 39(1) 45(1) 44(1) -21(1) -2(1) -20(1)

C(9) 40(1) 54(2) 39(1) -20(1) -1(1) -23(1)

C(10) 63(2) 58(2) 52(1) -20(1) -1(1) -27(1)

C(11) 81(2) 69(2) 111(2) 1(2) -30(2) -21(2)

C(12) 132(3) 73(2) 85(2) -33(2) -10(2) -50(2)

C(13) 35(1) 53(1) 40(1) -17(1) -3(1) -21(1)

C(14) 53(2) 55(2) 38(1) -20(1) -1(1) -24(1)

C(15) 43(1) 54(2) 44(1) -21(1) 1(1) -21(1)

C(16) 68(2) 64(2) 56(2) -20(1) -8(1) -13(2)

C(17) 99(3) 69(2) 75(2) -11(2) -14(2) 1(2)

C(18) 101(3) 57(2) 108(3) -27(2) -2(2) -8(2)

C(19) 85(2) 71(2) 94(2) -49(2) 2(2) -23(2)

C(20) 53(2) 64(2) 58(1) -31(1) 0(1) -23(1)

_______________________________________________________________________

Table 5. Hydrogen coordinates ( x 10^4) and isotropic displacement parameters (A^2 x 10^3) for 6n.

________________________________________________________________

x y z U(eq)

________________________________________________________________

H(1A) 6419 2653 2414 74

H(2A) 7207 525 1810 100

H(3) 5740 396 588 109

H(4) 3486 2371 -4 101

H(5) 2741 4502 585 74

H(7A) 3491 6022 1359 56

H(7B) 4865 5256 2161 56

H(9) 1233 4693 5317 49

H(10) 2253 2602 6883 67

H(11A) 4938 1114 6703 140

H(11B) 4708 2649 6803 140

H(11C) 5329 2250 5507 140

H(12A) 3381 2014 4496 135

H(12B) 1629 2293 5205 135

H(12C) 3041 891 5724 135

H(14A) 1790 5842 8400 56

H(14B) 124 5716 8930 56

H(16) -282 8144 6139 79

H(17) -1457 10646 5743 115

H(18) -1943 11815 7265 118

H(19) -1286 10472 9199 98

H(1) 4278(14) 4590(20) 4200(20) 78(8)

H(2) 233(19) 5170(20) 7164(17) 54(6)

________________________________________________________________

Table 6. Torsion angles [deg] for 6n.

________________________________________________________________

C(6)-C(1)-C(2)-C(3) -0.3(4)

C(1)-C(2)-C(3)-C(4) -0.4(4)

C(2)-C(3)-C(4)-C(5) 0.9(4)

C(3)-C(4)-C(5)-C(6) -0.8(4)

C(2)-C(1)-C(6)-C(5) 0.3(3)

C(2)-C(1)-C(6)-C(7) 177.65(19)

C(4)-C(5)-C(6)-C(1) 0.2(3)

C(4)-C(5)-C(6)-C(7) -177.13(19)

C(1)-C(6)-C(7)-C(8) -92.7(2)

C(5)-C(6)-C(7)-C(8) 84.6(2)

C(9)-N(1)-C(8)-O(1) 7.4(3)

C(9)-N(1)-C(8)-C(7) -169.67(16)

C(6)-C(7)-C(8)-O(1) -76.9(2)

C(6)-C(7)-C(8)-N(1) 100.2(2)

C(8)-N(1)-C(9)-C(13) -128.45(19)

C(8)-N(1)-C(9)-C(10) 107.2(2)

N(1)-C(9)-C(10)-C(11) 60.2(2)

C(13)-C(9)-C(10)-C(11) -64.2(2)

N(1)-C(9)-C(10)-C(12) -65.4(2)

C(13)-C(9)-C(10)-C(12) 170.26(18)

C(14)-N(2)-C(13)-O(2) -5.9(3)

C(14)-N(2)-C(13)-C(9) 172.39(19)

N(1)-C(9)-C(13)-O(2) -27.1(3)

C(10)-C(9)-C(13)-O(2) 97.7(2)

N(1)-C(9)-C(13)-N(2) 154.58(18)

C(10)-C(9)-C(13)-N(2) -80.6(2)

C(13)-N(2)-C(14)-C(15) 89.6(2)

N(2)-C(14)-C(15)-C(16) -3.4(3)

N(2)-C(14)-C(15)-C(20) 176.00(19)

C(20)-C(15)-C(16)-C(17) 1.7(4)

C(14)-C(15)-C(16)-C(17) -178.9(2)

C(15)-C(16)-C(17)-C(18) -0.1(4)

C(16)-C(17)-C(18)-C(19) -0.7(5)

C(17)-C(18)-C(19)-C(20) -0.2(5)

C(18)-C(19)-C(20)-C(15) 1.9(4)

C(18)-C(19)-C(20)-Cl(1) -178.2(2)

C(16)-C(15)-C(20)-C(19) -2.6(3)

C(14)-C(15)-C(20)-C(19) 177.9(2)

C(16)-C(15)-C(20)-Cl(1) 177.47(17)

C(14)-C(15)-C(20)-Cl(1) -2.0(3)

________________________________________________________________

Symmetry transformations used to generate equivalent atoms:

Table 7. Hydrogen bonds for shelxl [A and deg.].

____________________________________________________________________________

D-H...A d(D-H) d(H...A) d(D...A) <(DHA)

N(1)-H(1)...O(2)#1 0.876(9) 2.099(10) 2.967(2) 171(2)

____________________________________________________________________________

Symmetry transformations used to generate equivalent atoms:

#1 -x+1,-y+1,-z+1
